# Diverging prefrontal cortex fiber connection routes to the subthalamic nucleus and the mesencephalic ventral tegmentum investigated with long range (normative) and short range (ex-vivo high resolution) 7T DTI

**DOI:** 10.1007/s00429-021-02373-x

**Published:** 2021-09-05

**Authors:** Volker A. Coenen, Máté D. Döbrössy, Shi Jia Teo, Johanna Wessolleck, Bastian E. A. Sajonz, Peter C. Reinacher, Annette Thierauf-Emberger, Björn Spittau, Jochen Leupold, Dominik von Elverfeldt, Thomas E. Schlaepfer, Marco Reisert

**Affiliations:** 1 Department of Stereotactic and Functional Neurosurgery, Medical Center of Freiburg University, Breisacher STraße 64, 79106 Freiburg, Germany; 2 Medical Faculty of Freiburg University, Freiburg, Germany; 3 Department of Diagnostic and Interventional Radiology, Medical Physics, Medical Center, University of Freiburg, Freiburg, Germany; 4 Center for Deep Brain Stimulation, Medical Center of Freiburg University, Freiburg, Germany; 5 Anatomy and Cell Biology, Medical School OWL, Bielefeld University, Bielefeld, Germany; 6 Fraunhofer Institute for Laser Technology (ILT), Aachen, Germany; 7 Institute of Forensic Medicine, Medical Center of Freiburg University, Freiburg, Germany; 8 Division of Interventional Biological Psychiatry, Department of Psychiatry and Psychotherapy, Medical Center of Freiburg University, Freiburg, Germany; 9 Laboratory of Stereotaxy and Interventional Neurosciences, Department of Stereotactic and Functional Neurosurgery, Medical Center of Freiburg University, Freiburg, Germany; 10 Institute for Anatomy and Cell Biology, Department of Molecular Embryologie, Faculty of Medicine, Freiburg University, Freiburg, Germany

**Keywords:** dMRI, Deep brain stimulation, Hyperdirect pathways, Anterior limb of internal capsule, Major depression, Obsessive compulsive disorder, Superolateral medial forebrain bundle

## Abstract

Uncertainties concerning anatomy and function of cortico-subcortical projections have arisen during the recent years. A clear distinction between cortico-subthalamic (hyperdirect) and cortico-tegmental projections (superolateral medial forebrain bundle, slMFB) so far is elusive. Deep Brain Stimulation (DBS) of the slMFB (for major depression, MD and obsessive compulsive disorders, OCD) has on the one hand been interpreted as actually involving limbic (prefrontal) hyperdirect pathways. On the other hand slMFB’s stimulation region in the mesencephalic ventral tegmentum is said to impact on other structures too, going beyond the antidepressant (or anti OCD) efficacy of sole modulation of the cortico-tegmental reward-associated pathways. We have here used a normative diffusion MRT template (HCP, *n* = 80) for long-range tractography and augmented this dataset with ex-vivo high resolution data (*n* = 1) in a stochastic brain space. We compared this data with histological information and used the high resolution ex-vivo data set to scrutinize the mesencephalic tegmentum for small fiber pathways present. Our work resolves an existing ambiguity between slMFB and prefrontal hyperdirect pathways which—for the first time—are described as co-existent. DBS of the slMFB does not appear to modulate prefrontal hyperdirect cortico-subthalamic but rather cortico-tegmental projections. Smaller fiber structures in the target region—as far as they can be discerned—appear not to be involved in slMFB DBS. Our work enfeebles previous anatomical criticism and strengthens the position of the slMFB DBS target for its use in MD and OCD.

## Introduction

Direct connections of the human cortex with subcortical structures are of special interest for the regulation of behaviour. Since the early days of psychiatric surgery it has been known that these subcortical pathways can be targeted with strategic lesioning approaches (Spiegel et al. 1947) to alter pathological states and most of this information is a result of the meticulous workup of surgical cases (Meyer et al. 1947; Meyer 1949; Beck 1950; Spiegel et al. 1951). Later, more sophisticated staining techniques were used on postmortem brains to visually distinguish fibers of passage from actually severed fibers (Nauta 1958; Cowan et al. 1972; Nauta and Gygax 2009). Deep Brain Stimulation (DBS) belongs to the standard regimen in the treatment of medically treatment resistant neuropsychiatric disorders especially of movement disorders (Coenen et al. 2015; Lozano et al. 2019). Since the late 1990s DBS has been investigated for obsessive compulsive disorders, Tourette syndrome and later for major depression (Larson 2008). In none of these psychiatric indications it has so far become a standard of care although for OCD it might be regarded as emerging therapy (Wu et al. 2020). In the search for an alternative target for treatment resistant depression (TRD), a dMRI derived tractographic target was introduced, the slMFB (superolateral branch of the medial forebrain bundle) (Coenen et al. 2011). As a direct connection between the prefrontal cortex and subcortical structures (through the anterior limb of the internal capsule) like the nucleus accumbens (NAC) and mesencephalic ventral tegmentum (MVT) it appeared to be a promising candidate for DBS antidepressant efficacy. The structure was thoroughly characterized (Coenen et al. 2009, 2012) before it was introduced in single center clinical trials (Schlaepfer et al. 2013; Fenoy et al. 2016, 2018; Coenen et al. 2019a). Starting with the work of Haynes et al. (Haynes and Haber 2013) some silent criticism against the slMFB and especially against its nomenclature emerged (Haber et al. 2020). Their work on comparative macaque anatomy used sophisticated tract tracing cortical injections and defined a limbic hyperdirect pathway (lHDP) a structure which is now by some authors understood and positioned as the commonly stimulated fiber tract involved in DBS for OCD (Li et al. 2020; Haber et al. 2020). On a closer look, the original lHDP description is not fully in line with the strict definition of a hyperdirect pathway especially since part of the limbic connections to the STN are vastly unclear; Haynes and Haber defined a limbic STN cone to explain the part of their traced fibers which terminated in the lateral hypothalamus, medial and outside the STN. In doing so, they appear to ignore any direct access of the PFC to the MVT (Wu et al. 2013), e.g., a PFC to MVT feedback pathway (Oades and Halliday 1987). However, a predescribed fiber termination field in the VTA (An et al. 1998; Öngür et al. 1998; Price 2006) is potentially identical with this limbic STN cone. It is a region which we and others have successfully stimulated in the discussed indications with the DBS technology in clinical trials (Schlaepfer et al. 2013; Fenoy et al. 2018; Coenen et al. 2019a). A direct MVT access is moreover found in dMRI tractographic aggregation studies on OCD (Smith et al. 2020; Li et al. 2020) and furthermore in connectomic studies of the STN (Isaacs et al. 2018; Temiz et al. 2019). For various reasons the tract found in these aggregation studies in our eyes shares many features reminiscent of the slMFB (with a direct access to MVT) rather than of a lHDP, as these studies interpret. Tract tracing information in non-human primates point towards clearly segregated lateral routes of hyperdirect projections (Nambu et al. 1996, 1997; Coudé et al. 2018) while medial routes have been described with overlapping regions of orbitofrontal cortex (OFC) injections (An et al. 1998; Öngür et al. 1998; Schmahmann and Pandya 2006; Frankle et al. 2006). We speculate that in the (lateral) human OFC at least two networks (Coenen et al. 2020) interact: (a) the reward network, projecting over a medial route into the MVT, largely confluent with the slMFB; (b) the control network which projects to the subthalamic nucleus and beyond via a lateral trajectory. Obviously, there is a fundamental disagreement concerning a potential functional and anatomical disentanglement of these lateral and medial pathways which finds its expression in conflicting concepts (lHDP vs. slMFB).

We initiated this study and used distinctive methods (long-range normative dMRI, short range high resolution ex-vivo 7T dMRI and histology) to further investigate the perceived contradiction: We aimed at separating fiber connections of the frontal cortex to the midbrain into lateral and medial projection routes. We further investigated the cortical origin of medial (MVT) and lateral (STN) projections both on a normative group level (HCP, *n* = 80) and in our post-mortem specimen (*n* = 1) in MNI space. Finally, we investigated the involvement of small mesencephalic fiber tracts in cases from two previous depression trials of slMFB DBS (*n* = 24).

## Methods

### Data

**Fig. 1:**
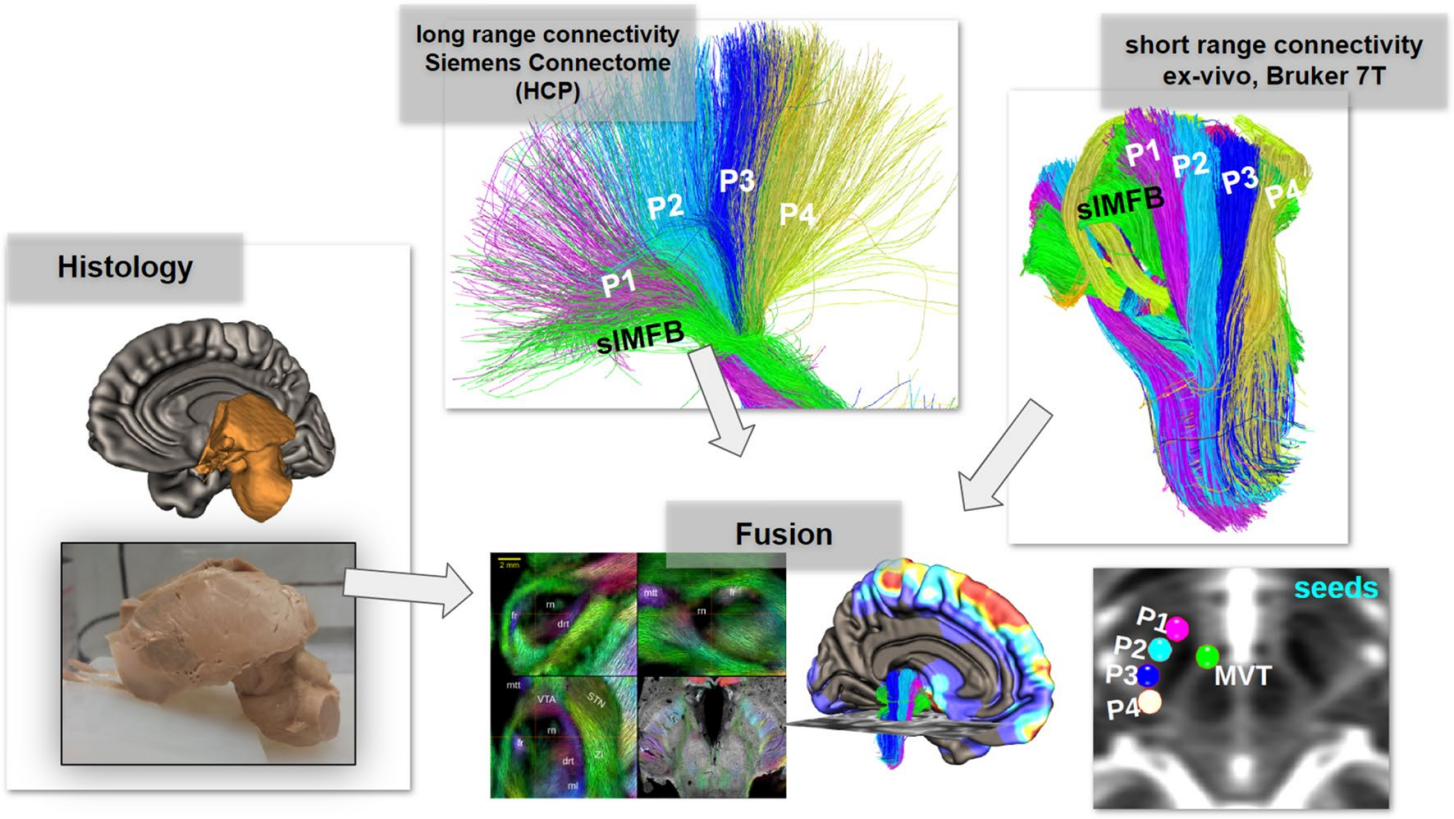
Overview of the techniques utilized in this contribution. Long-range (HCP) and short range (ex-vivo high resolution) DTI are integrated to serve with histological comparison. Lower right, seed regions (spheres) defining distinct tracts (P1–P4) of the cerebral peduncle and the mesencephalic ventral tegmentum (MVT)

We shortly depict the general approach (Fig. 1) and go into detail later. Basically, three types of data are merged: a group average dMRI connectome based on high quality HCP data, a highly resolved dMRI dataset from a single post-mortem brain, and histological micrographs (with TH and Luxol stainings) from the same individual. The postmortem dMRI is semi-automatically registered by a deformable warp to MNI space, where also the HCP group connectome lives. To understand long-range connectivity a whole brain connectome is reconstructed from the average HCP dMRI data. On the other hand, to detail out the short range connectivity a high resolution midbrain connectome is reconstructed from the postmortem sample. Finally, various selection criteria for streamlines traversing the anterior and posterior internal capsule are applied.

### Human brain specimen

The donated brain was obtained via a collaboration with the University of Freiburg, Department of Anatomy, and the histological assessment was carried out with the full consent of the local ethical committee (Ethics: The use of human brain tissue was granted by the IRB of Freiburg University under no. 203/11). The specimen is from an adult female over 80 years of age who donated her body for research. She had no known neurological medical history or pathology.

The process of body fixation/ brain ex-plantation was done according to the Department of Anatomy’s standard protocols. In short, complete body fixation was performed by perfusion with formaldehyde through the femoral arteries (3.7% formaldehyde) and the intact brain was extracted and stored in a container in 1% formaldehyde/2% 2-phenoxyethanol solution. Prior to histological processing, the arachnoid membrane/ meninges and vessels were removed, the brain was cut, photographically documented and scanned in a small animal MRI scanner.

### Postmortem MR imaging

Postmortem scans were made with a 7T Bruker Biospec 70/20 preclinical scanner with a bore diameter of 200 mm, obtained with a linear 1H, manual tuning and matching volume coil from RAPID Biomedical with an internal diameter of 72 mm. Diffusion data of the brainstem were acquired using Bruker's 3D spin-echo DTI sequence in three thick slabs: the first two with echo time (TE) = 30 ms, repetition time (TR) = 310 ms, FOV = 50 × 50 × 20 mm, matrix size = 240 × 240 × 27, resolution = 0.208 × 0.208 × 0.741 mm, and scan time with each slab of 28 h 27 min. Due to timing issues, the third slab was acquired with partly different parameters: echo time (TE) = 30 ms, repetition time (TR) = 240 ms, FOV = 50 × 50 × 20 mm, matrix size = 240 × 240 × 27, resolution = 0.208 × 0.208 × 0.833 mm, and scan time was of 19 h 35 min. All three slabs were imaged using 1 b0 image and 50 diffusion directions (*b-*value = 8000 s/mm^2^).

### Postmortem histology (embedding, slicing and staining)

The brain sample (preperated and reduced in size, Fig. 1) was placed in a customized Perspex box (20 × 20 × 20 cm), with a titanium base and four mountable/removable walls (see Appendix Fig. 17). Approximately 2 L of a 5% agar solution was poured into the box, and when it started to set, the brain was placed on the agar base and the anterior commissure—posterior commissure was horizontally aligned. When the agar base hardened with the brain in the desired position, the rest of the 8 L of the agar was gently poured into the box to completely embed the brain. The agar was allowed to set overnight at room temperature, and the two walls in the longitudinal axis were removed and exchanged with walls with railings every 5 mm to permit the precise and guided slicing of the brain using a blade (Autopsie Klingen, Pfm-medical, #205000325-325 mm). By fixing and sliding the blade in the rails on the opposite walls, 5-mm axial macro slides of the entire brain were made. The macro sections were photographed, and kept at 4 °C in 30% glucose/PBS solution to cryo-protect the tissue. Selected macro sections were cleaned of the surrounding agar, embedded in Tissue Tek, and cut at −40 °C using a microtome. The serial 40 µm sections were collected in PBS (1 section/ well in a 6-well plate) and kept at 4 °C.

Sections were mounted on large chrome-alum coated slices (76 × 52 mm, Carl Roth^®^) and air-dried overnight. Tyrosine-Hydroxylase (TH): sections were washed three times using PBS and quenched by incubating for 10 min in 3% H_2_O_2_ and 10% methanol, washed with PBS and placed in blocking solution (3% BSA; 0.03% Triton X-100) for 2 h. The edges around the slides were dried, and using a hydrophobic pen (PAP pen, Abcam-ab2601) a barrier was drawn around the section to limit the required antibodies used. Sections were incubated with the primary antibody in a humidity chamber overnight (1:500, rabbit anti-TH, Merck #AB152). The next day the slides were washed with in PBS, exposed to the secondary antibody in the humidity chamber for 1 h (1:200, anti-rabbit IgG-biotinyl, DAKO E0432), and incubated in an avidin–biotin-solution for another hour (ABC Elite; Vector Laboratories, Burlingame, CA). The color reaction was induced with 3,3′-diaminobenzidine (DAB) and 0.01% H_2_O_2_. Sections were washed, mounted, dehydrated and cover slipped prior to analysis. Luxol-Blue (modified Klüver-Barrera protocol): Sections were dehydrated and placed in Luxol Blue solution (0.1% Luxol blue/0.1% Cresyl violet/0.05% Lithium carbonate/1% Acetic acid) for 12 h at 37 °C, washed in 70% ethanol, and ddH_2_O, and left in lithium carbonate for 2 min. Sections were washed in ddH_2_O, and left in Cresyl violet for 20 min, and washed in acetic acid until the desired color intensity was obtained. The sections were dehydrated, and covered slipped prior to analysis.

Finally, the stained sections were digitized using a digital microscope/slide scanner (Pannoramic Desk II DW, 3DHistech Ltd) and the image analysis was performed with the software Caseviewer 2.2. (3DHistech) and converted to Nifti (a tomographic imaging format) to allow registration with the MRI.

### HCP diffusion MRI

The dMRI data for long-range display are based on the Human Connectome Project database (https://ida.loni.usc.edu/login.jsp)). We used data from 80 subjects from the Human Connectome Project (Q1:S3) data corpus (resolution 1.25 mm isotropic, three b-shells with 1000, 2000, 3000, for more details on the protocol and preprocessing see (Glasser et al. 2013)). For all subjects the warp to MNI space is computed via CAT12, which uses the T1-weighted image (http://dbm.neuro.uni-jena.de/cat12/CAT12-Manual.pdf in the Statistical Parametric Mapping software (SPM12, www.fil.ion.ucl.ac.uk/spm/software/spm12).

Prior to reconstruction of the whole brain group connectome, the raw diffusion data (dMRI) are warped to MNI space using the CAT12 warps and averaged. To account for the diffusion gradient orientations during averaging, the reorientation of the local diffusion directions is based on the Jacobian matrix (Raffelt et al. 2012). The spatial resolution of the dMRI data in template space is 1.5 mm isotropic.

### Tractography

For both, the whole brain HCP tractography, and the short range post-mortem sample the global approach (GT) from (Reisert et al. 2011) is used. As opposed to local walker-based tractography, global fiber tracking tries to find a fiber configuration that best explains the acquired diffusion weighted MRI data. Practically, the optimization process is similar to a polymerization process, where initially the streamlines are short and fuzzy, while during optimization connections proliferate and fibers become more and more congruent with the data. We used the Diffusion & Fibertools toolbox, which is publicly (http://www.uniklinik-freiburg.de/mr-en/research_groups/diffperf/fibertools.html) available and implements the algorithm proposed in Reisert et al. (2011). Details and parameters are given below.

### Postmortem MRI processing

Prior to tractography, the dMRI data were denoised using random-matrix theory (Veraart et al. 2016) and upsampled to a resolution of 0.208 × 0.208 × 0.370 mm by an edge-preserving upsampling scheme (Reisert and Kellner 2017). For quality control standard tensor fitting procedure was applied: fractional anisotropy values are in the range of 0.3–0.6 in regions of high anisotropy (like the cerebral peduncle). Mean diffusivity values of the sample are in the range of 0.15–0.25 µm^2^/ms. A brain tissue mask was manually constructed by thresholding and manual refinement. Global tractography was performed using the following parameters: segment length: 600 µm, segment width: 200 µm, iterations: 10^9^. The weight parameter was automatically adapted according to the GT toolbox. The obtained tractogram has a size of approximately 300,000 streamlines. To obtain higher robustness the tracking was repeated five times and merged. The computation time was approximately 24 h. Based on the tractogram, high resolution tract density images were computed at an isotropic resolution of 200 micron as well as color-coded tract densities.

### HCP processing

As already detailed above the normalization of the dMRI data to MNI space was performed by CAT12 together with a proper reorientation and interpolation of the gradient directions. Based on the dMRI data in template space, global tractography was applied using the default ‘dense’ parameter set and used ten re-iterations to gain a robust number of estimated fiber bundles (Schumacher et al. 2018). As reconstruction area the white matter segmentation obtained from CAT12 at the loose threshold of 0.5 was used.

### Registration HCP/postmortem/histology

Ex-vivo MR contrasts differ substantially from in-vivo MR contrasts, which complicates the registration and matching between each other. We found that global tracking derived fiber densities are indeed quite similar in contrast, and hence, we used them for registration using ANTS (https://www.nitrc.org/projects/ants) in combination with a manual refinement step. The refinement step comprises the definition of corresponding landmarks in both spaces. For the identification of landmarks in the postmortem sample we exclusively used the tract densities. In MNI space we additionally used high-resolution T1/T2-weighted contrast for the localization. Landmarks included the positions of the fasciculus retroflexus, the nucleus ruber and anterior commissure. For registration of the 2D histological slices to the postmortem MR, it is not possible to use the MNI space as a reference, because it is not possible to apply a deformable 3D warp onto a non-tomographic 2D imaging slice. However, to stay as close as possible to MNI space, we applied beforehand the purely affine part of the deformation. The actual registration mainly consisted of a manual search for the translational parameter. An accurate registration was not possible due to several disruptions of the histological specimen.

### Tract selection

For the *virtual* resection of bundles (as a set of streamlines) from the HCP whole brain tractography, and postmortem midbrain tractogram, respectively, we used mostly spherical volumes in MNI space coordinates. In Appendix Fig. 16 and Table 1 we report the coordinates (selection templates) for all considered structures. Central to our work is the subdivision of the cerebral peduncle into four parts (P1–P4) and the waypoint through the ventral tegmentum (VTA). A selection template consists of a central, rather tight, bottleneck, defining the character of the structure, and two rather broad waypoints, which are used to avoid streamlines crossing the central seed in perpendicular directions. While the streamlines passing P1–P4 appear to be rather unimodal (one prominent bundle), the streamlines passing through the VTA seed (−6.0, −12.0, −8.0) can be decomposed into three further subdivisions. Thus, we added here additional waypoints for distinction. For the mesolimbic pathway we added one small and restrictive waypoint medial to the RN and, correspondingly, lateral to the RN for the mesocortical pathway. The motoric and sensory connections of the VTA (Hosp et al. 2019) are captured by an adaption of the posterior waypoint. For the analysis of the cortical projection patterns of the HCP long-range connectivity (VTA and P1–P3), we did not use the terminal broad waypoints to avoid any bias. As one additional structure, which is not covered by Table 1 we used the STN as a seed region for the HCP long-range connectivity (template taken from Ewert et al. 2018).

### Bundle-specific refinement

To remove spurious streamlines and visualize the dissected bundles in an appealing way, we used the following refinement step: Each bundle is rendered back into a tensor-valued volume, where tangents of the streamlines are additively accumulated as rank-1 tensors. Each tensor volume represents the local bundle density together with its main local orientation, and can be used in a straightforward manner for ordinary streamline tractography (like in bundle specific tractography, Rheault et al. 2019). The threshold of the tracking mask was chosen such that singled out spurious fibers are not included. For streamline seeding 500 random voxels within the upper 20%-percentile of the fiber density were chosen.

### Long-range cortical projections

**Fig. 2:**
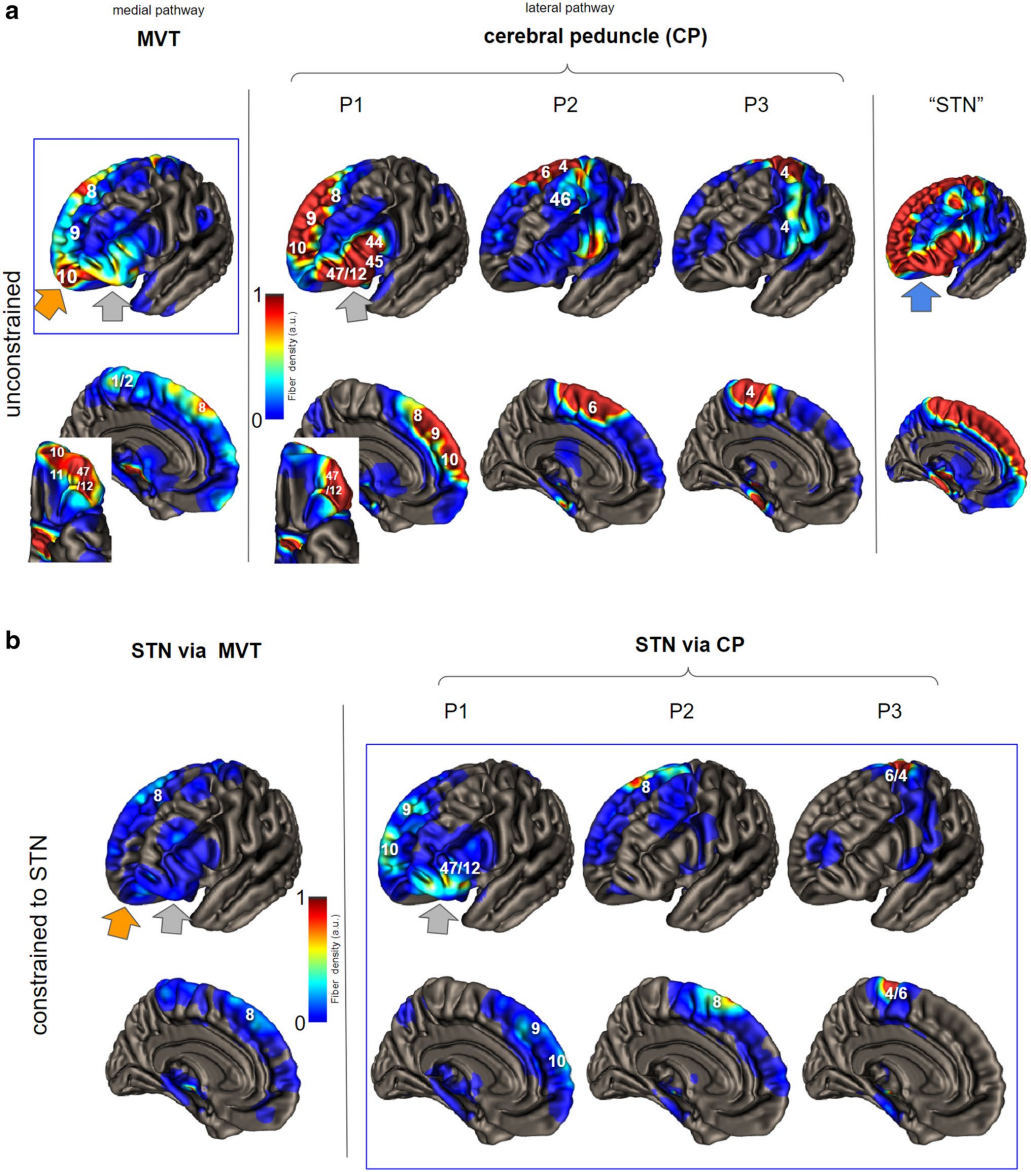
Frontal cortical origins of projections to the midbrain derived from HCP template (*n* = 80) indicating some cortical overlap but not congruence. Numbers in image refer to Brodman’s regions (Brodmann 1909) *Upper panel*, unconstrained fibers to ventral tegmentum (MVT) and three different parcellations of the cerebral peduncle (CP: P1–P3, for seed regions cf. Fig. 1 C, P4 here left out). Some notable overlap is found between MVT (seed) and P1 (seed) in DLPFC and lateral OFC (grey arrow) as well as STN. STN, cortical projection origin to the entire STN, which comprises essentially the sum of MVT and P1-P3. Note how the orbital cortex (blue arrow) contributes to “STN” when not constrained via lateral fiber routes. *Lower panel*, fiber projections through CP (P1–P3) but now constrained to the subthalamic nucleus (STN). Note dramatically reduced fiber connections of PFC/OFC to MVT when constrained to STN. STN shows clearly less PFC but more motor connections. In comparison (blue rectangles) of VTA and P1, the frontopolar region (orange arrow) exclusively obtains unconstrained MVT fibers (P1, lateral OFC, BA 47/12; VTA, medial OFC and frontopolar, BA10). Some overlaps between MVT and STN fibers (blue rectangle) are seen in BA 9, BA10 (although different parts) and BA 47/12

To understand the cortical projective fields of the cerebral peduncle P1–P3, VTA and STN we computed streamline terminal densities and visualize them on the gray matter surface (we used T1_IXI555_MNI152_GS surface template from CAT12). To account for uncertainty and gray matter coverage, we smoothed the terminal density by a Gaussian of width 4 mm. All terminal densities are displayed in an identical color scaling (see Fig. 2).

## Results

The main results are summarized in the figure legends (Figs. 1, 2, 3, 4, 5, 6, 7, 8, 9, 10, 11, 12). In principle an integration of long-range and short-range tractography is feasible, allowing to investigate long-range connectivity while at the same time benefiting from the high resolution capacities in the ex-vivo sample.

**Fig. 3:**
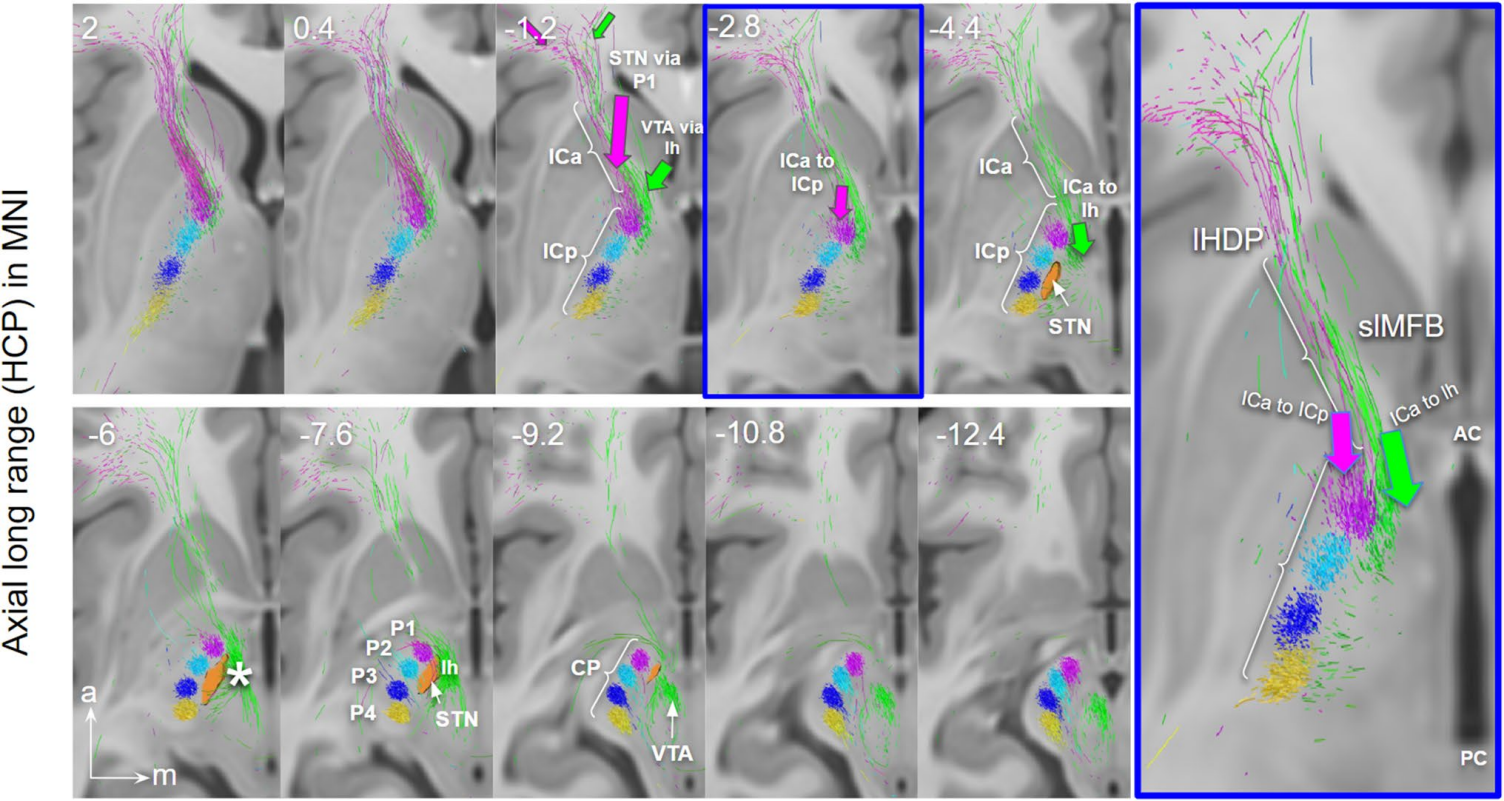
Long-range HCP depiction of PFC fibers connecting to STN (via CP) and ventral tegmentum (via lh), axial view. Fibers reaching the STN are forced via cerebral peduncle thus entering the midbrain region from later. Fibers reaching the ventral tegmentum are routed over the lateral hypothalamus. STN fibers change from the anterior limb of internal capsule (ICa) to posterior limb (ICp) just above the anterior commissure (purple arrow) to reach the STN from the lateral. VTA fibers leave ICa (at level of AC, green arrow) to traverse the lateral hypothalamus (lh) and in the midbrain are located medially to the STN. *STN* subthalamic nucleus; *VTA* ventral tegmental area; *VTA termination field; *ICa* internal capsule, anterior limb; *ICp* internal capsule posterior limb; *CP* cerebral peduncle; *P1*–*P3* major fiber routes of the cerebral peduncle; *P1 *fronto-pontine tract; *P2* cortico-bulbar tract; *P3* cortico-spinal-tract; *P4* occipito-temporo-pontine tract; *lHDP* limbic hyperdirect pathway; *slMFB* superolateral branch of the medial forebrain bundle

**Fig. 4:**
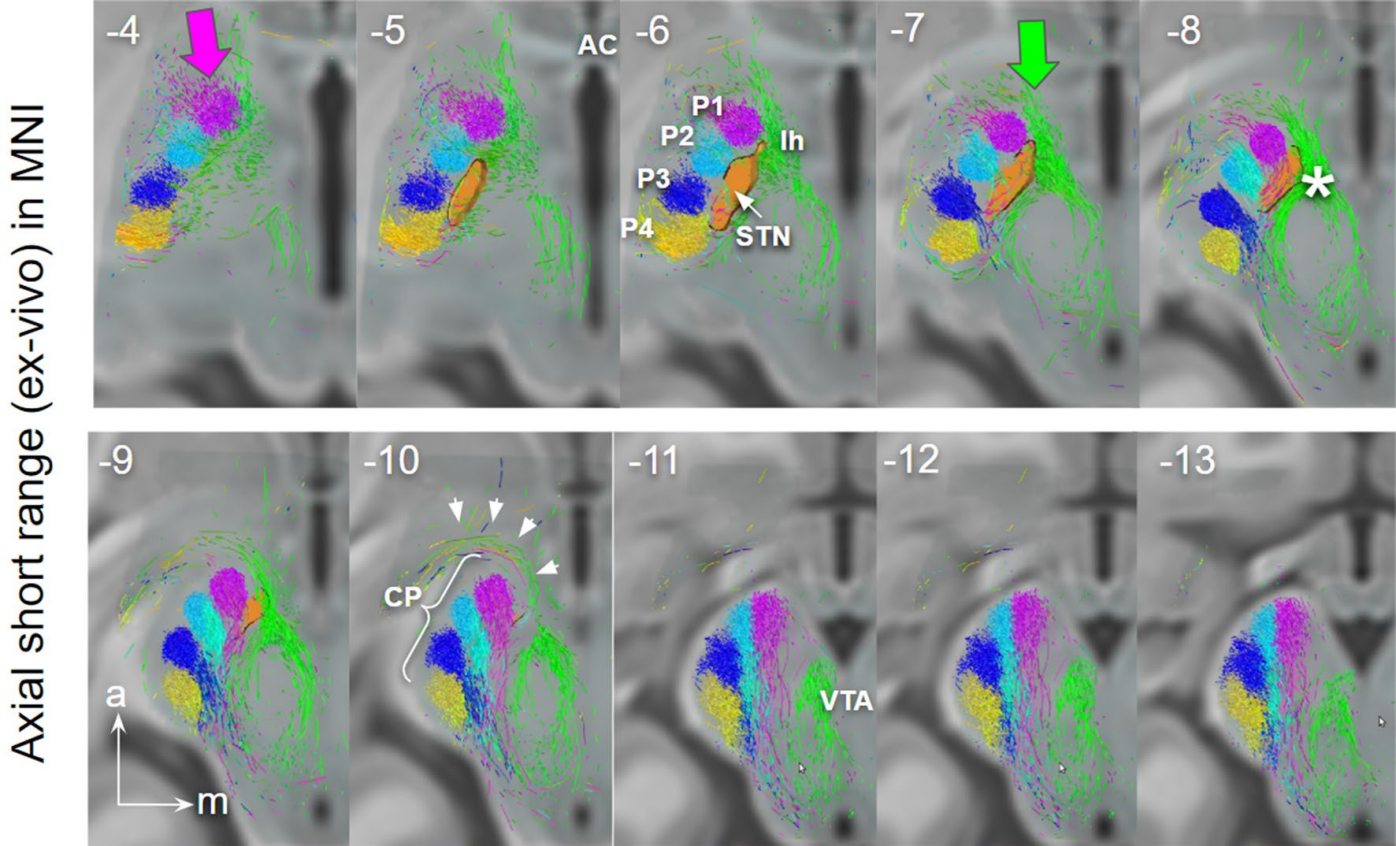
Short range ex-vivo depiction of PFC fibers connecting to STN and ventral tegmentum. Long-range situations can be verified. Due to simple and unconstrained selection criteria additional small fiber tracts (e.g., ansa lenticularis, white arrows) are picked up. (For legend cf. Fig. 3, *VTA termination field of PFC fibers)

**Fig. 5:**
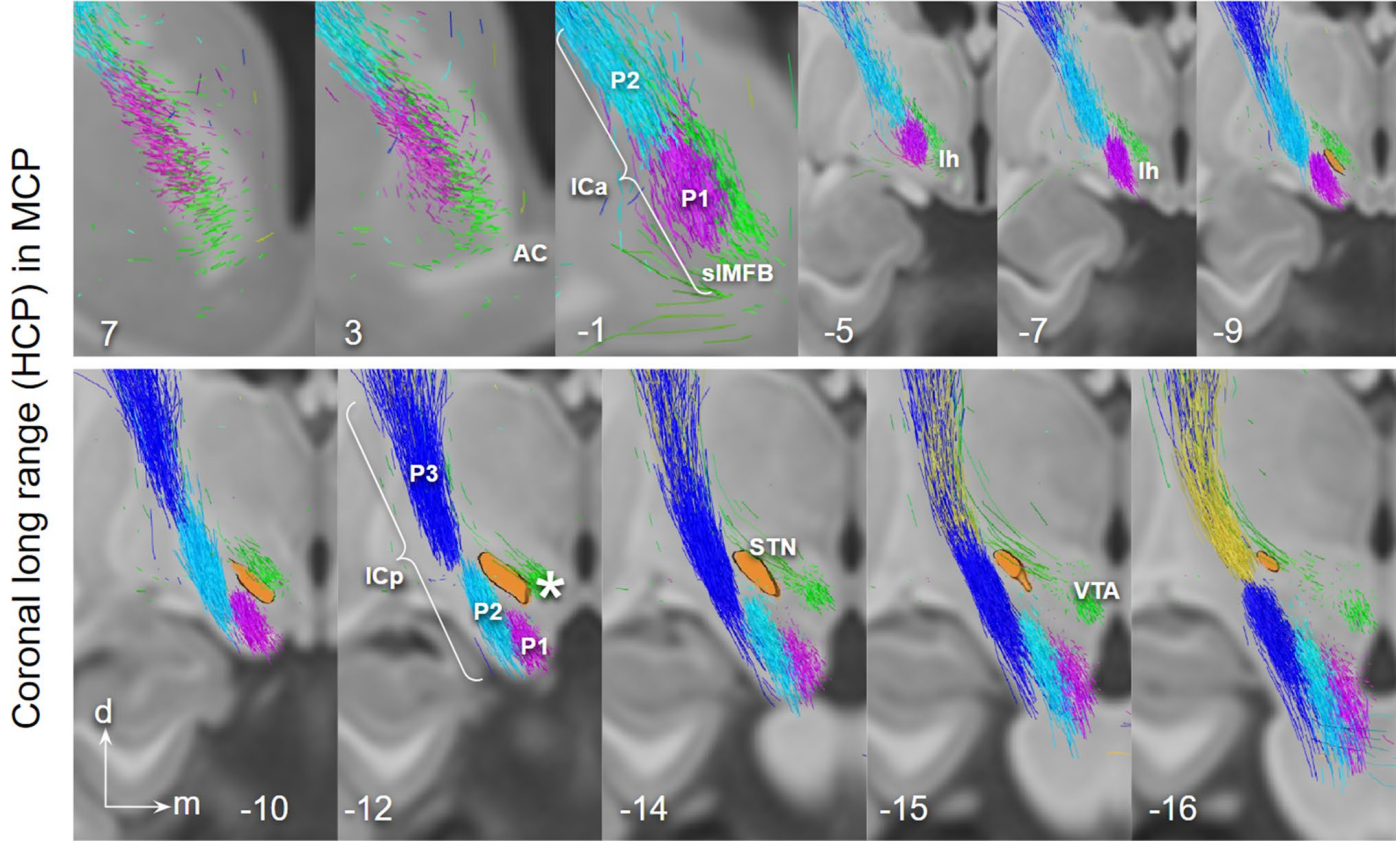
Long-range HCP depiction of PFC fibers connecting to STN (via P1–P3) and ventral tegmentum (via lh), coronal view. Note how fibers from VTA end most ventrally in ICa. (Legend: cf. Fig. 4.)

**Fig. 6:**
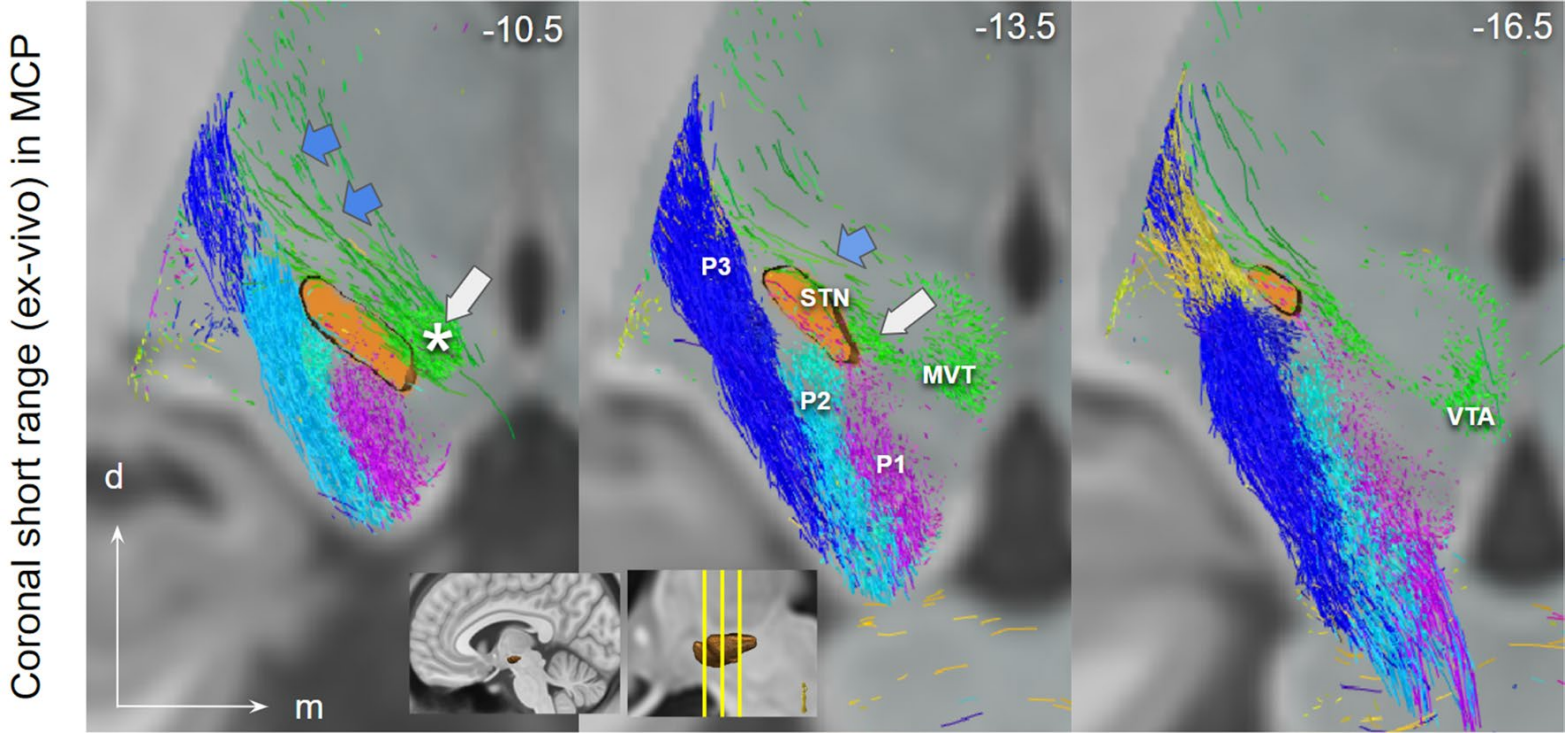
Short range ex-vivo depiction of PFC fibers connecting to STN (via CP, P1–P3, lateral) and ventral tegmentum (via lh, medial), coronal view. Close proximity of P1–P3 with STN. P1 fibers traverse the nucleus. White arrow marks the region in MVT/PRF identical with “limbic STN cone” in Haynes and Haber (2013). Blue arrows point to motorMFB fibers in Zi. Insets show the position of coronal cuts. *Zi* zona incerta; *MVT* mesencephalic ventral tegmentum; *VTA* ventral tegmental area; * VTA terminal field (approximated))

**Fig. 7:**
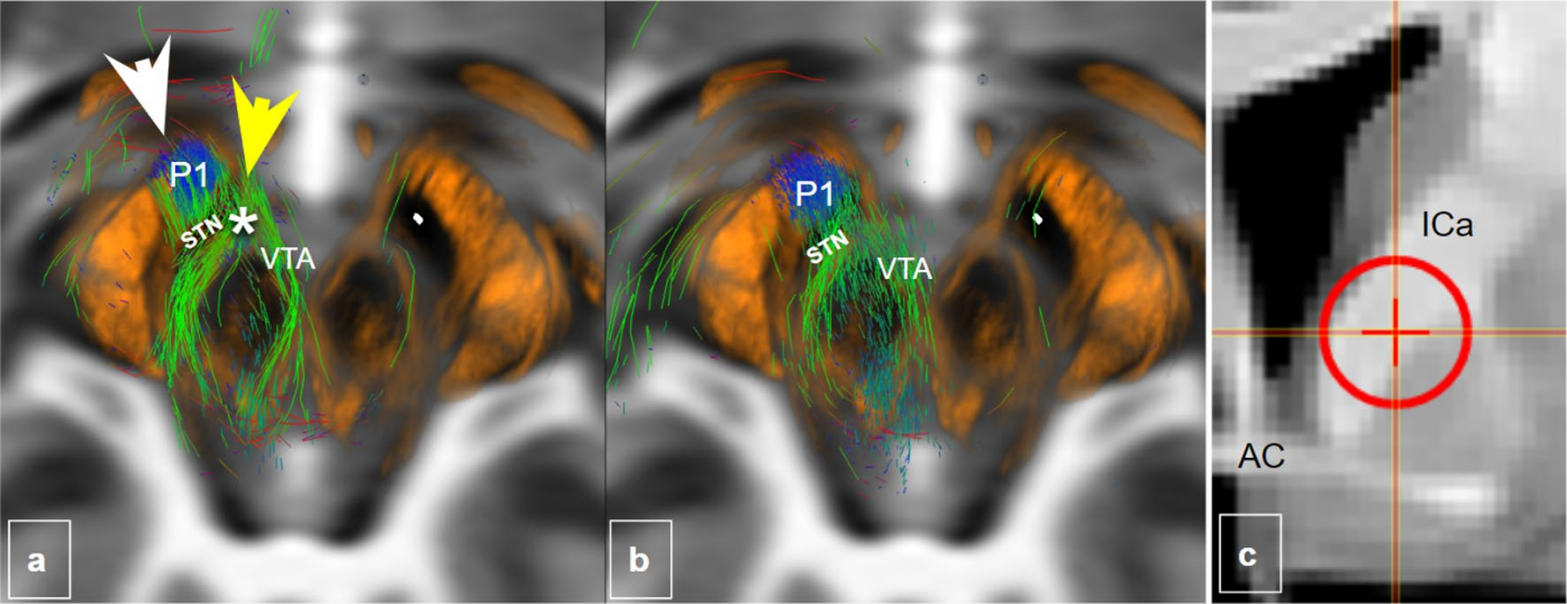
Fiber selection cross-check from ICa. **a**, **b**, axial midbrain sections showing joint streamline depiction of fibers medially (VTA, green; yellow arrow–head) and laterally (P1, frontopontine tract, blue, white arrow head) to the STN when selecting from a lower position in the anterior limb of the internal capsule (ICa) at the level of the anterior commissure (AC)

**Fig. 8:**
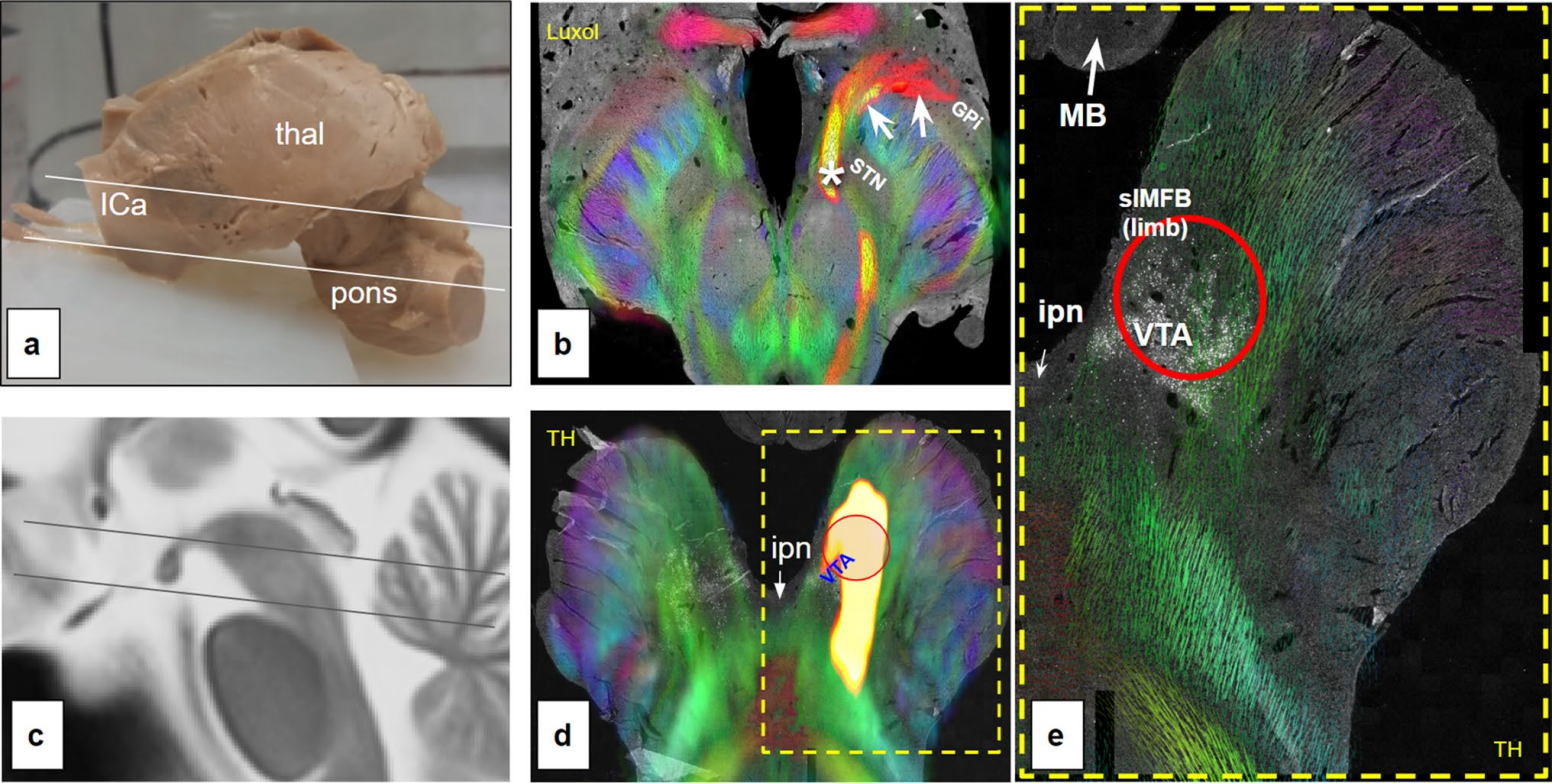
Visualization of ex-vivo (*n* = 1) combined 7T DWI measurement and histological analysis. Tractography of slMFB (yellow) is based on transmitter-information from thyroxine-hydroxylase (TH) staining and the identified VTA cell group. **a** Ex-vivo specimen; **b** midbrain section with color-coded directional information (green, anterior–posterior; blue, superior-inferior; red, medial–lateral) superimposed on histological slice (luxol fast blue in black/white). Note nice alignment between DTI and histology information including rendition of “Edinger’s comb” (cerebral peduncle), white arrows indicate ansa lenticularis. **c** MNI brain midline indicating superior (**b**) and inferior cuts (**c**); **d**, similar as **b** but more inferior. TH staining shows Dopamine cells of ventral tegmental area (VTA); red circle serves as seed region for slMFB (yellow/red). **e** Magnification from **d**. *ICa* anterior limb of internal capsule; *thal* thalamus; *TH* tyrosine hydroxylase; *MB* mammillary body; *ipn* interpeduncular nucleus; *STN* subthalamic nucleus; *Gpi* globus pallidus internus; *VTA termination field of PFC fibers (approx.)

**Fig. 9:**
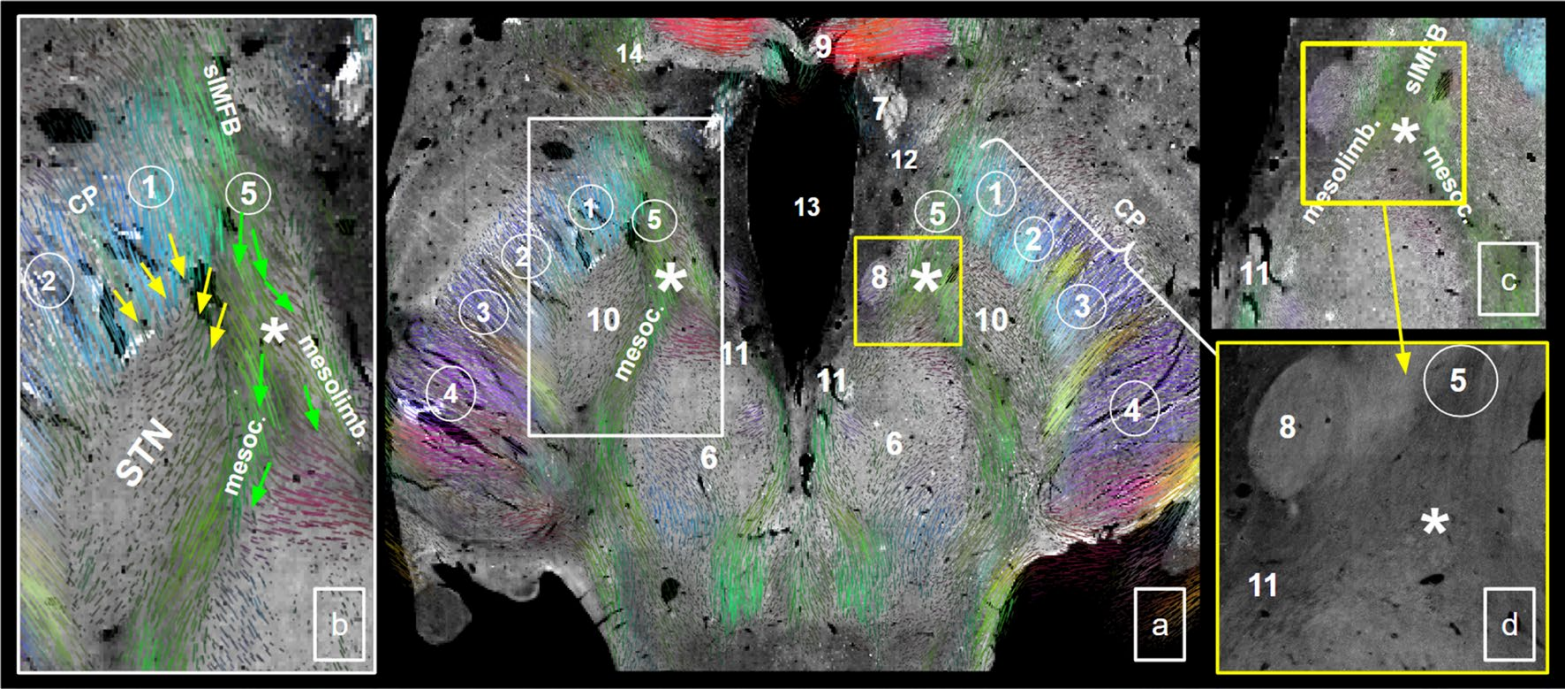
Midbrain ventral tegmentum and adjacent hypothalamus and cerebral peduncles from short range ex-vivo DWI. **a** Analysis of DWI-information (including color coded directional and in-plane directional information). slMFB enters the ventral tegmental midbrain through posterior lateral hypothalamus (5) to curve towards VTA (11). **b** fibers from CP (P1&P2) enter STN at its tip and from lateral. slMFB fibers pass medially to STN and follow, on a separate route, to VTA (11). **c** confluens fibers and slMFB. **d** histological slice (luxol fast blue shown in b/w) shows a similar situation. 1, frontopontine tract; 2, corticobulbar tract; 3, corticospinal tract, 4, occipito-temporo-pontine tracts; 5, entry of slMFB into ventral midbrain; 6, red nucleus; 7, fornix; 8, mammillothalamic tract; 9, anterior commissure; 10, STN; 11, ventral tegmental area (of Tsai); 12, lateral hypothalamus; 13, third ventricle; 14, anterior commissure; CP, cerebral peduncle; slMFB, superolateral medial forebrain bundle; messolimb., mesolimbic fibers; mesocort., mesocortical fibers; * VTA termination field/medial STN region; *STN* subthalamic nucleus

**Fig. 10:**
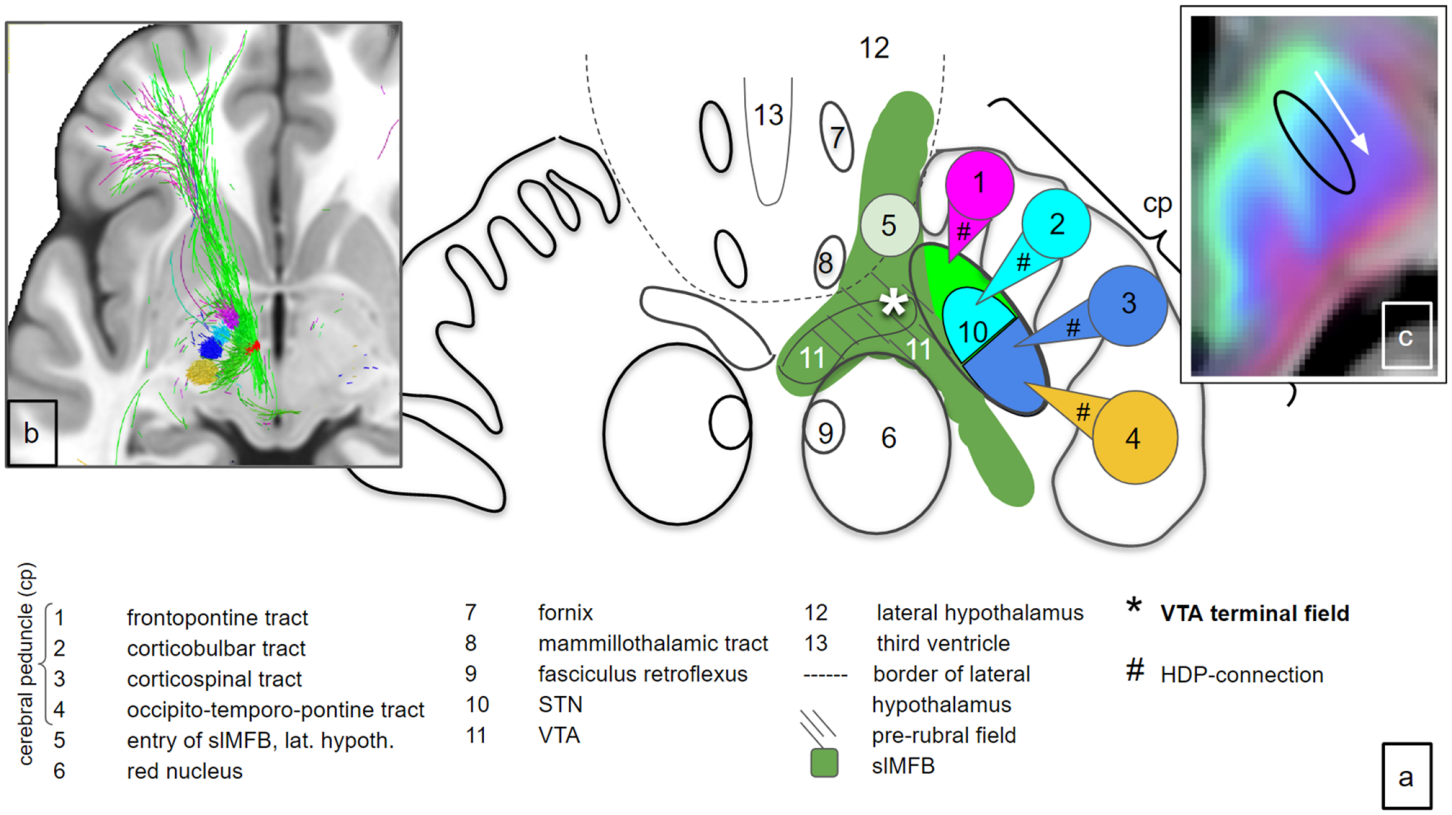
Midbrain ventral tegmentum and adjacent hypothalamus and cerebral peduncles. **a** Cartoon rendition of organisational principle as proposed here: subthalamic nucleus receives connections from cortical regions via hyperdirect projections (#) out of the cerebral peduncle (lateral). Note the extension of the VTA towards the white matter between the red nucleus and STN in analogy to (Trutti et al. 2021). *VTA terminal field of descending OFC fibers (potentially identical with “medial STN region”, “limbic STN cone”) in principle only reached via slMFB which reaches the midbrain through lateral hypothalamus. **b** long-range implementation. **c** SPECTRE rendition taken from (Reisert et al. 2021)

**Fig. 11:**
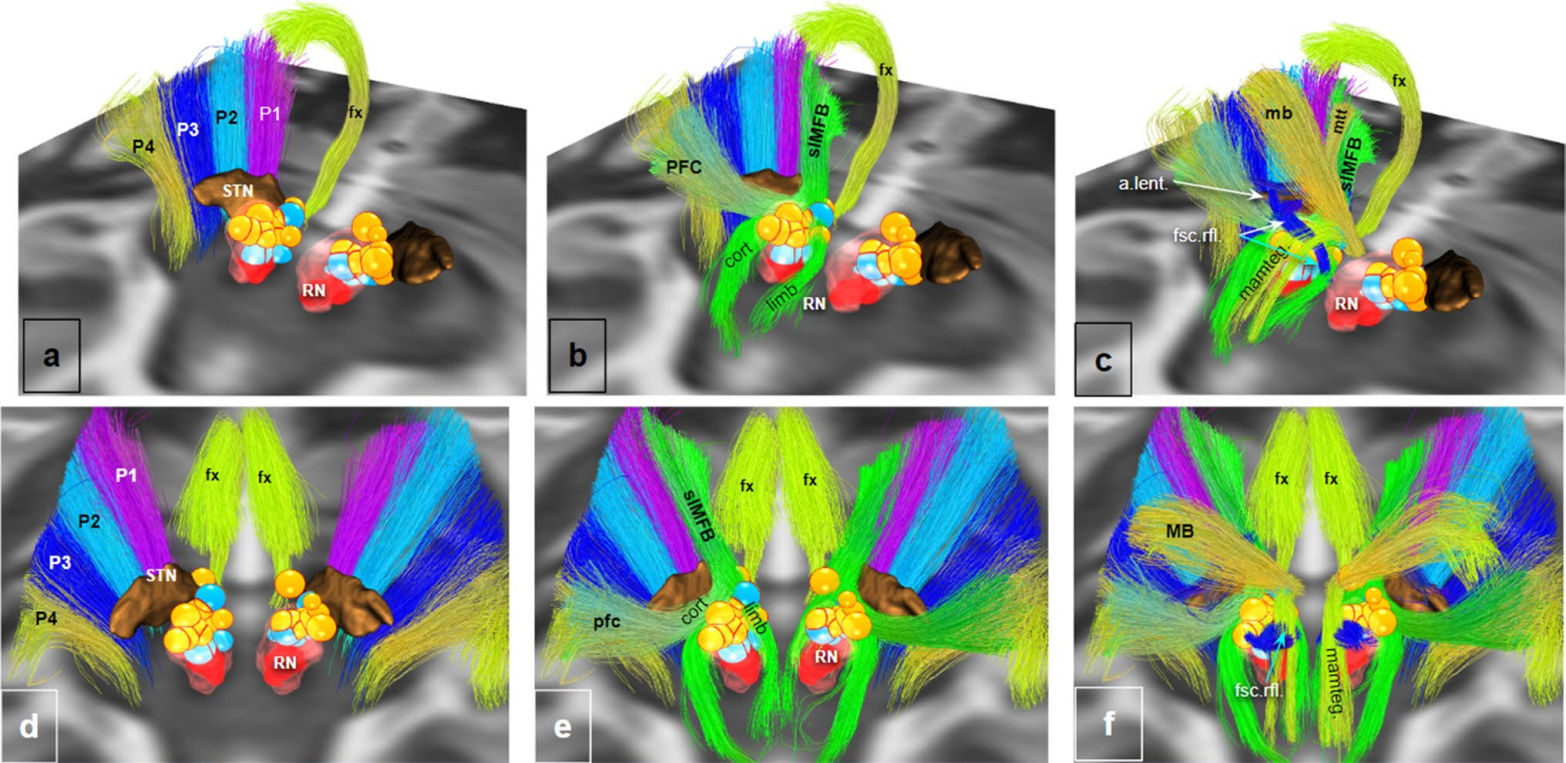
Midbrain integrative views of large (P1–P4, slMFB) and small fiber tracts in MNI space (ex-vivo DTI). Concentration of volume of tissue activation spheres of responders (yellow) and non-responders (blue) in the pre-rubral field of the MVT. The Dichotomy between lateral (P1–P4) and medial pathways (slMFB) is clearly demonstrated. **a**–**c**, view from posterior and right. **d**–**f**, view from superior. Yellow spheres represent simulated activation volumes from responders in FORESEE and FORESEE II trials for treatment-resistant major depression (Deep Brain Stimulation of the superolateral medial forebrain bundle). For response- and simulation-criteria cf. (Coenen et al. 2019b). Note: Responder and non-responder spheres aggregate on slMFB without any significant contact to smaller pathways and STN. *P1* fronto-pontine tract; *P2* cortico-bulbar tract; *P3* cortico-spinal-tract; *P4* occipito-temporo-pontine tract; *slMFB* superolateral branch of the medial forebrain bundle; *cort* mesocortical fibers, limb, mesolimbic fibers; *MVT* mesencephalic ventral tegmentum: pfc, prefrontal cortex pathway (motor MFB); *mb* mammillary body pathway (motor MFB); *mtt* mammillo-thalamic tract; *fx* fornix; *mamteg* mammillo-tegmental tract; *a. lent* ansa lenticularis; *fsc. rfl.* fasciculus retroflexus (of Meynert))

**Fig. 12:**
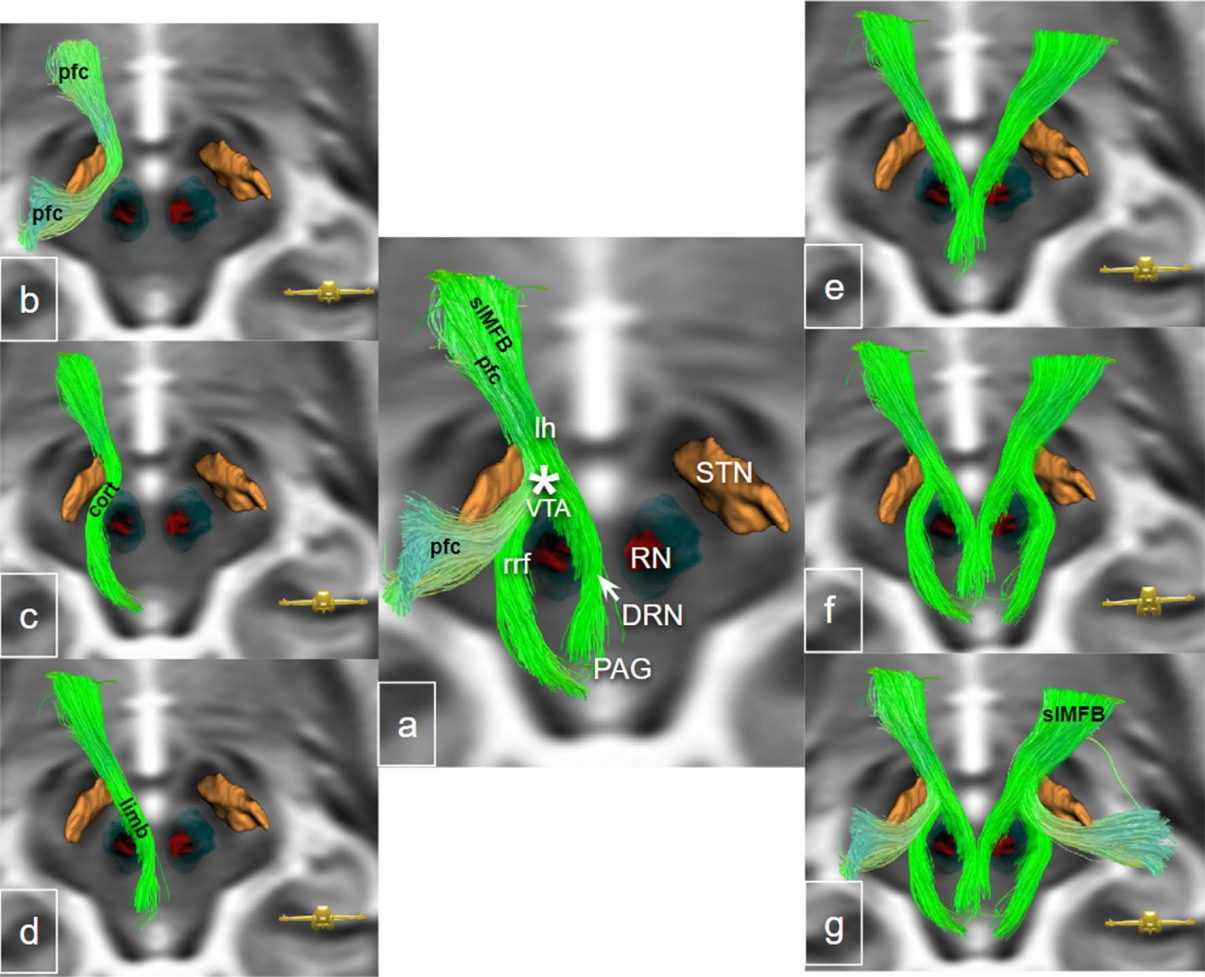
Midbrain representation of the slMFB including parts of the motor MFB (pfc bundle) as previously described in Hosp et al. (2019). **a** Fiber structures shown on the left, nuclei on the right. pfc and slMFB as part of the greater MFB (Fig. 13) commingle. The cortical and limbic parts of the slMFB nicely follow the ventral tegmental area (VTA) and connect to the dorsal raphe nucleus (DRN) and retrorubral fields (rrf). **b**, **c** Distinction between pfc (prefrontal cortex to motor bundle of motor MFB) and slMFB subparts as described in **a**. **e**–**g** Bilateral representation of fibers. * VTA terminal field (estimated); *VTA* ventral tegmental area; *rrf* retro-rubral field; *pfc* prefrontal cortex bundle of the motor MFB; *cort* potential mesocortical fibers; *limb* potential mesolimbic fibers; *RN* red nucleus; *DRN* dorsal raphe nucleus; *PAG* periaqueductal grey; *STN* subthalamic nucleus; *lh* lateral hypothalamus; *slMFB* superolateral branch of the medial forebrain bundle

### Cortico-subcortical projections

The results are summarized in Fig. 2 (also see Fig. 15 Appendix). The typical “global” cortical projections are found when not constraining to lateral or medial routes. STN is reached by a gradient of fibers from OFC, frontopolar, superior frontal gyrus, motor region, and inferior frontal gyrus. When constraining STN fibers via their physiological lateral route (for seed regions cf. Fig. 1, seeds) the pattern is essentially reduced to frontal eye fields (BA8), dominantly motor regions (BA4,6) and the lateral orbitofrontal and inferior frontal (BA47/12) region. OFC fibers are largely eliminated. Constraining cortico-mvt projections via STN essentially erases the entire connection.

### Lateral and medial frontal projection routes in HCP and ex-vivo

The presence of distinct corticofugal fiber pathways to the midbrain is plausible: (a) the slMFB to the mvt which shows a cortical connection largely from Brodman’s area **10, 11**, 12/47 but also 8 and 9; (b) the hyperdirect pathways (HDPs) to the subthalamic nucleus with a distinct projection pattern from BA **6, 8**, 9, 10, (24), **44, 45, 46**, **12/47.** BA10 principally projects to the VTA and only minimally to the STN. We were able to identify two pathways—one lateral one medial—with only partly overlapping cortical fields of origin (in part dlPFC and lOFC). Both fiber pathways travel in parallel through the ICa (anterior limb of the internal capsule) as a pass through (Coenen et al. 2020). The reward related fibers from medial and central OFC (**BA10 and 11**) are located at the most ventral part of the ICa. The two pathways diverge at the level of the anterior commissure. Medial reward related fibers (slMFB) from here take a trans-hypothalamic route and enter the mesencephalic ventral tegmentum medial to CP and STN through the posterior and lateral hypothalamus (Fig. 3). They appear to traverse the lateral VTA and here separate in a medial (to VTA) and a lateral (to retro-rubra fields) part. Connections from MVT to the STN are unclear. The HDP fibers are confluent but lateral and superior to slMFB fibers in the ICa (Fig. 5). HDP fibers (esp. P1) change their position from ICa to ICp to reach the STN exclusively via a lateral route out of the cerebral peduncle.

### Cross-checking fiber routes

Seeding from the most ventral part of the ICa (Fig. 7) leads to addressing fibers which utilize both a medial (yellow arrow head in Fig. 7) and a lateral route (white arrow head in Fig. 7) to the midbrain. As a consequence, both fiber routes will therefore also be addressed when seeding from the STN (especially when it is artificially volume increase to show more fibers in its vicinity) (cf. Fig. 3, upper panel). This can potentially lead to false positives with respect to STN fiber projections (again, as a rule HDP fibers in principle enter the STN from lateral).

### Visual inspection of DWI results in conjunction with histological specimen

A direct comparison of histological information with MRI (including anatomical, dMRI and tractography) was performed. A comparison with a fiber staining (Luxol) and staining for tyrosine hydroxylase (to find the dopaminergic cell groups of the VTA) is shown in Fig. 8. Here, the mesolimbic part of the slMFB is shown to be in spatial proximity to the VTA cells. Seeding from this region shows most parts of the slMFB and serves as confirmation for the slMFB’s relation to this DA cell group.

A detailed comparison is shown in Fig. 9. The direction encoding visualization of the short range (ex-vivo high resolution) DTI allows for a better understanding in conjunction with actual histology: Most parts of fiber tracts entering the MVT via the lateral hypothalamus (medial pathway) appear to pass by the subthalamic nucleus and continue to more dorsal regions, potentially the retro-rubral field (mesocortical). On the other hand, some fiber bundles pass medial to proceed to the VTA. The lateral pathways—here largely presented as columns in the cerebral peduncle (1–4)—subserve the STN with hyperdirect collaterals. Edinger’s comb (Horn et al. 2019) shows nicely in this depiction. Figure 10 conceptualizes this idea and further shows extension of the VTA.

### Investigation of small fiber tracts in the midbrain

Figure 11 summarizes the results of the projection of responder/non-responder volume of tissue activations onto a single high resolution data set in MNI space. Connectomic information about the analyzed responders/non-responders has been published in Coenen et al. (2019b). As a brief repetition, the fiber tractographic analysis in this previous work was not able to differentiate responders from non-responders. In our analysis here, responder- and non-responder spheres concentrate on the larger targeted fiber tract, the slMFB without a regular co-stimulation of smaller fiber tracts. Figure 12 introduces the slMFB and (part of the motorMFB) once more for a better overview and distinction to Fig. 11.

## Discussion

We will in the following address parts of the midbrain as the mesencephalic ventral tegmentum (MVT, hatched region in midbrain inset in Fig. 14). The MVT is the anterior part of the tegmentum (thus excluding cerebral peduncles and the tectum) and anatomically *contains* the pre-rubral field (as an anatomical description for the region just in front and below the red nucleus) and the larger parts of the ventral tegmental area (VTA) of Tsai.

We have here researched the hypothesis that the mesencephalic ventral tegmentum (including PRF and VTA) and the subthalamic nucleus (STN) are sub-served by distinct but confluent and only partially overlapping fiber projections from the prefrontal cortex. For this purpose, we have used a new technique, namely an integration of long-range normative DTI and short range high resolution ex-vivo DTI together with histological information, and made them available in a common stochastic space (MNI brain). We have routed tractographic algorithms to follow either medial or lateral fiber projections on their way to the midbrain and looked in detail at the resulting cortical connectivity patterns. Furthermore, we have selected image data of responders and non-responders from two DBS depression trials (FORESEE I & II) and simulated patient individual volume of tissue activation models into our long-range/short range integrative MNI fiber model. We further scrutinized the target region of slMFB DBS for the presence of distinct small fiber tracts (others than slMFB), possibly causative for an antidepressant effect during stimulation (DBS) of this region.

### STN and hyperdirect pathways

Cortico—STN connections are termed hyperdirect pathways (HDPs) and have important functions in motor control but also in non-motor regulation (Nambu et al. 2002; Temel et al. 2005; Mosley et al. 2019; Chen et al. 2020; Polyakova et al. 2020).

With respect to motor control, these connections were described initially with autoradiography in the macaque (Monakow et al. 1978). With the use of intracortical microstimulation—guided anterograde tracing experiments, Nambu et al. found M1 input predominantly in the lateral part of the STN while SMA inputs predominantly reached the medial nucleus (Nambu et al. 1996, 1997). Single axon tracing experiments recently revealed details about the principle course of hyperdirect connections from the motor cortex (Coudé et al. 2018). This work described HDP entry into the STN nucleus from its most dorsal tip (lateral). A thin myelinated (Mathai et al. 2013) collateral axon breaks off the main corticofugal projection (part of the internal capsule, IC) and enters the STN. It then builds further generations of collateralization to reach multiple STN neurons with axon terminals (Fig. 14). The HDP collateral neurons are not exclusively reaching the STN but on several instances exit the STN medially to continue to the zona incerta (ZI) or the red nucleus. Thus HDPs do not exclusively address the STN but also other structures in its medial proximity (Coudé et al. 2018). The function of these extended parts of the HDP is speculative. Our results here fit perfectly with the described organization principle of this lateral pathway. When cortical connections of the STN are regarded with dMRI tractography it makes sense to constrain these connections via a lateral route of the cerebral peduncle (CP, P1–P4).

#### Non-motor hyperdirect connections

In search for non-motor HDP connections to the STN, pertinent research in tract tracing studies failed to show a rich connection of the antero-medial STN to the OFC region (Haynes and Haber 2013) in the macaque monkey. Their descending fibers traveled in the IC. Typical HDP fibers branched off the brainstem IC (CP) to enter the STN lateral. For motor and cognitive (prefrontal) HDP, the authors found a similar organization pattern like previous studies (Nambu et al. 1996, 1997) with collateral HDP fibers traversing the STN from lateral to medial. However, vmPFC and OFC fibers coursed along the medial tip through the hypothalamus and terminated medial and outside the anterior STN a region which belongs to the lh or the ventral tegmentum (Fig. 14). The authors define this region as “limbic STN cone” (Haynes and Haber 2013) and functionally integrate this region into the limbic STN. Our results shed new light on these findings: Based on the trans-hypothalamic course and the termination field of these fibers it has to be debated if these fibers belong to the STN at all. It can—moreover—be reasoned, that the fibers to the region medial and outside of the STN are not representing a hyperdirect pathway in the classical sense (not from lateral, passage through the lateral hypothalamus, likely not myelinated). Moreover, the applied tract tracing methods are standard but have limitations: In an overlap of projection pathways, cortical injections will address neuronal populations of different systems (HDP but also others), potentially leading to ambiguous results (“limbic cone of the STN outside the STN in lh”). Previous work, applying similar anterograd techniques and similar injection regions surprisingly did not report PFC projections to the STN but found evidence for—albeit sparse—connections to VTA and SN (Frankle et al. 2006). These results are in keeping with work from other groups who in tract tracing experiments found trans-hypothalamic projections from the OFC to the MVT especially terminating in distinct columns of the periaqueductal grey (PAG) (An et al. 1998) or in the hypothalamus (Öngür et al. 1998). Moreover, *axons from the medial part of the OFC (mPFC) have been found to enter the midbrain and form a terminal field in the VTA before continuing to the MVT and the PAG* (An et al. 1998; Öngür et al. 1998; Price 2006). *Price formulates* “[…] it is likely that the cortical projections are a major pathway for forebrain modulation of bodily reactions.” and later “[…] it is characterized by outputs to the visceral control structures in the hypothalamus and brainstem, and is involved in cortical modulation of visceral functions. In addition, this system is involved in mood and emotional behaviour.” This interpretation points to a function in keeping with the slMFB. It is evident that these connections are made to GABA-ergic neurons and only secondarily reach VTA DA neurons (Tong et al. 1996). Fibers further ramify around serotonergic cells in the midline (dorsal raphe nucleus). On another note, it is clear that there are anatomical and functional discrepancies between the human and the macaque brain (Petrides et al. 2012; Wallis 2012) which can only to a certain extent be overcome. It is therefore questionable if an animal with certainly reduced emotional and cognitive capacities (compared to humans) can serve as a direct model for human anatomy and physiology, although the mere organizational principles of fiber orientation appear to be similar between species (Jbabdi et al. 2013). Our work confirms recent work on structural and cortical to subcortical DTI derived connectivity via the hyperdirect pathways which already showed only minimal connections to the limbic or medial STN region (Temiz et al. 2019). These authors also find a dichotomy between midline/medial and lateral (hyperdirect) projection. They suggested an intrinsic STN connection to the limbic STN subserved via a lateral route. They similarly have found a pathway to the mesencephalic ventral tegmentum as a source of limbic connections of this region to the PFC. Such intrinsic connections appear to be confirmed by literally all tract tracing studies which scrutinize the hyperdirect (albeit motor or supplementary motor) connections in the macaque (Coudé et al. 2018). The work of Isaacs et al. (2018) likewise underpins a reduced connection of the STN to regions involved in response inhibition. As such, our results of predominantly motor and premotor HDP to the STN are in line with these previous reports.

### Superolateral medial forebrain bundle

The slMFB, described already some time ago (Coenen et al. 2011, 2012), has recently been a topic of debate, potentially because its stimulation showed strong and long lasting antidepressant (Schlaepfer et al. 2013; Fenoy et al. 2016, 2018; Bewernick et al. 2017; Coenen et al. 2019a) and also anti OCD (Coenen et al. 2016) efficacy. The discussion which arose concerns mainly the nomenclature and not so much the existence of the pathway itself. On one hand, it represents a structure which is not described by classical anatomy (“[…] mismatch between the surgical target (slMFB) and anatomical literature (mfb) […]” (Li et al. 2020); “[…] not utilizing accepted anatomic structures or nomenclature […]” (Middlebrooks et al. 2020)) but on the other hand—and according to our judgement—emerges in several dMRI DBS aggregation studies as a resulting and causative structure for DBS efficacy (Smith et al. 2020; Vlis et al. 2020; Li et al. 2020) (see also discussion on pertinent DBS studies below). This latter circumstance obviously has the potential to leave some authors open to doubt and in troubled waters; while *some* neuroscientists have understood and adopted the concept and nomenclature (Bracht et al. 2014, 2015, 2019, 2021; Zacharopoulos et al. 2016; Denier et al. 2020; MacNiven et al. 2020; Fenoy et al. 2021) others feel the urge to find different names for a structure which in their eyes by no means can be akin to the mfb. This is insofar remarkable, since an anatomical similarity of the slMFB with the classical mfb has never been claimed (Coenen et al. 2012).

**Fig. 13:**
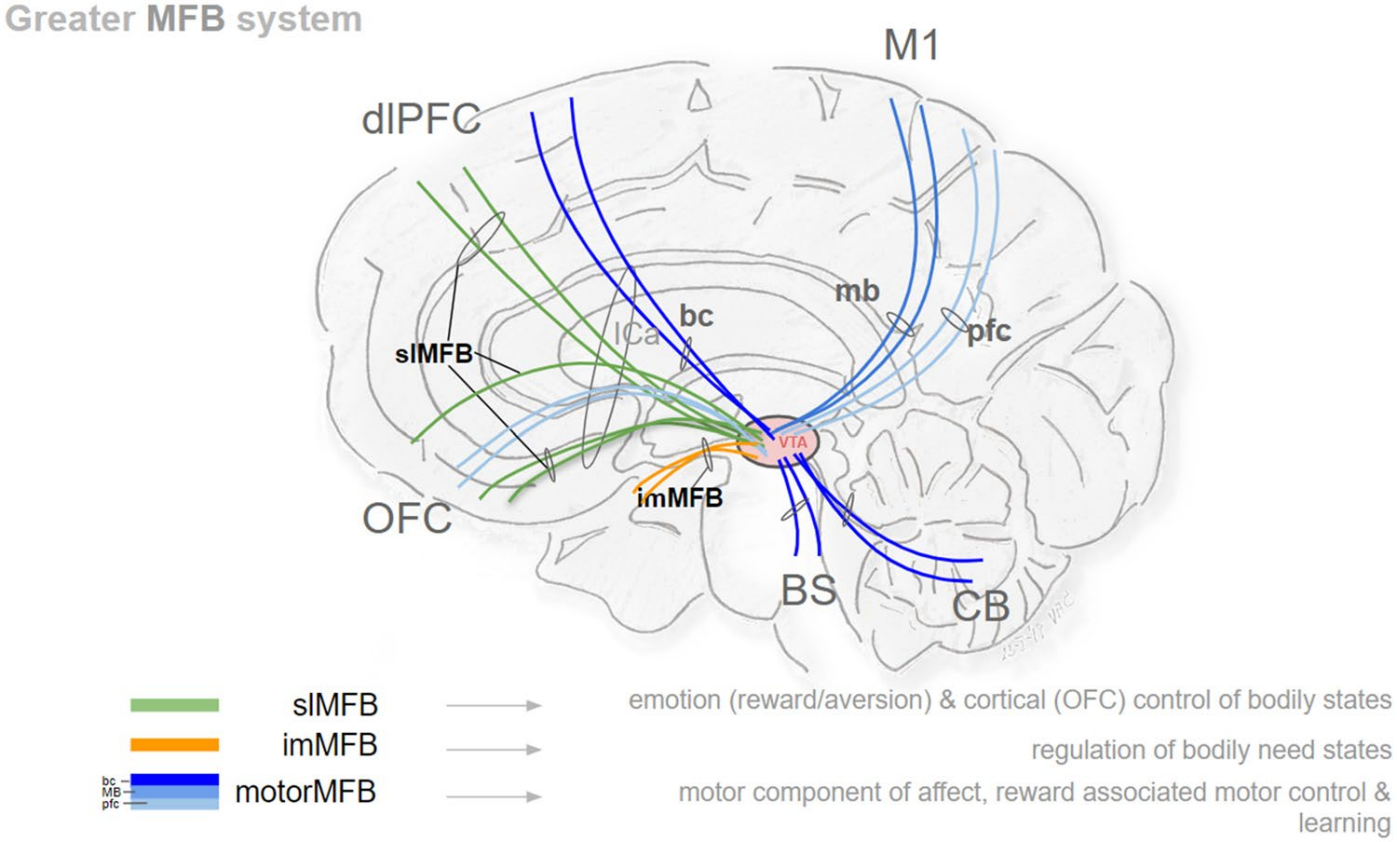
Greater MFB system (“the MFB”). Conceptual depiction. The MFB constitutes a system rather than a single pathway with multiple connections to and from the ventral tegmental area. Limbic parts are the slMFB and imMFB. The motorMFB consists of three sub-bundles (pfc, bc, mb). *slMFB* superolateral branch of the medial forebrain bundle (limbic); *imMFB* inferomedial branch of the medial forebrain bundle (limbic); *pfc* prefrontal cortex bundle (of motorMFB); *bc* brainstem/cerebellum bundle (of motorMFB); *mb* mammillary body bundle (of motorMFB); *M1* primary motor region; *dlPFC* dorsolateral prefrontal cortex; *OFC* orbitofrontal cortex; *BS brainstem*; *CB cerebellum*; *VTA* ventral tegmental area; *ICa* anterior limb of internal capsule

The slMFB is part of a greater MFB system with functions in emotion regulation (reward system), control of emotion associated bodily reactions, motor display of positive affect and motor learning (Fig. 13). MFB (in capital letters) is a conceptual term for a greater MFB system (hence subparts of it; imMFB, slMFB, motorMFB, Fig. 13) which serves to transport the mfb functions into a more complex framework of human primate anatomy (Coenen et al. 2020).

We will in the following use the abbreviation “mfb” for the classical description, which mainly arises from rodent anatomy. The mfb (rodent) is a bidirectional structure and besides the fornix and the stria terminalis is one of the strongest midbrain connections of the forebrain. It is a structure close to the midline of the brain (lateral hypothalamus). Most of the research on the mfb has been performed on rodents (Nieuwenhuys et al. 1982; Veening et al. 1982; Geeraedts et al. 1990a, b) and results were—as typical for comparative anatomy—transferred to human anatomy (Nieuwenhuys et al. 2008). This does not at all belittle this pioneering work, but shows the limitations this approach might bear. The mfb carries a plethora of transmitters. According to our understanding, the slMFB (as part of the MFB system)—like the rodent mfb—has to be bi-directional (Fenoy et al. 2021), but does not necessarily carry Dopamine to cortical levels (Oades and Halliday 1987). Dopamine (DA) as a main transmitter in our understanding is not a prerequisite for a structure to be called mfb/MFB. The mfb had been described many years (Herrick 1924; Nieuwenhuys et al. 1982) before the pioneering work by Dahlström, Fuxe (Dahlstroem and Fuxe 1964) and Ungersted (Dahlström et al. 1964; Andén et al. 1966; Ungerstedt 1971). They described for the first time monoaminergic cell groups (including NA and DA) and their projection routes (also via the mfb) with stereotactic accuracy. It is our understanding that the slMFB will likely NOT transport DA all the way through the striatal cleft or to cortical regions (Oades and Halliday 1987; Ciliax et al. 1999) although we regard the slMFB as bidirectional (Coenen et al. 2020). However, as a contributor to the greater MFB-system’s function the slMFB has influence on the midline DA pathways (imMFB) by utilizing slMFB’s corticofugal glutamatergic (Glu) projections to the midbrain tegmentum and VTA (Ikemoto 2010; Schlaepfer et al. 2013). As such, the slMFB suffices to act as part of a circuit system and feed back loop to the ventral tegmental area (VTA) (Oades and Halliday 1987). The Glu descending projections appear to play an important role in the enhancement of DA transmission with DBS to the mfb in rodents (Vajari et al. 2020). This Glu mechanism during slMFB DBS—amongst others—might play an important role for the antidepressant effect. It has recently been corroborated that a superior trajectory of the MFB exists (MacNiven et al. 2020) and potentially serves to fulfill the *ascending spiral hypothesis* of midbrain to forebrain connection and regulation (Haber et al. 2000; MacNiven et al. 2020).

The cortical projection to the ventral tegmental midbrain—the slMFB—is as a consequence of the interpretation of our work *not* part of Arnold’s bundle (prefronto-pontine tract, P1) which is a myelinated pathway, located lateral to the SNr as part of the cerebral peduncle and subserves like most parts of the CP the STN with myelinated (Mathai et al. 2013) HDP fibers. The slMFB is more likely a sparsely myelinated fiber tract consisting of a trajectory of short fibers. In the ICa’s ventral part, it is located lateral and most ventral even inferior to HDP fibers. The slMFB has an aslant and in larger parts trans-hypothalamic (Neudorfer et al. 2021) course as previously described (Coenen et al. 2011, 2012, 2018b). This trans-hypothalamic route before bridging to the midbrain in the prerubral field (with formation of a VTA terminal field before continuing to other parts of the MVT) makes the analogy to the classically and in rodents described mfb complete. This description further takes functional necessities into account which arise from the phylogenetically evolving human PFC which needs access to the limbic midbrain (Nauta 1972). This was the intention of the chosen nomenclature as slMFB (Coenen et al. 2011, 2012) in the first place for this largely overlooked fiber projection. *As we have said: An anatomical similarity with the classical mfb has never been claimed.*

### Mesencephalic ventral tegmentum and the limbic midbrain

The neuroanatomist Walle Nauta was already in the 1970s aware of divergent and parallel projections from the PFC to distinct target regions, including the STN and the ventral tegmental midbrain. He writes: “[…] the frontal lobe has been found to emit fibers to the striatum, to the subthalamic region, to a region of the mesencephalic tegmentum lateral and dorsal to the red nucleus, and to a medial zone of the pontine grey matter.” (Nauta 1972). This description intriguingly suffices the definition of the HDP and a parallel slMFB. With unspecific degeneration (after ablation) descending tracts from the PFC to the midbrain have been demonstrated in humans and monkey species (Meyer et al. 1947; Meyer 1949; Beck 1950; Vito and Smith 1964). Projections from the OFC to the mesencephalic tegmentum in the marmoset have likewise been described (Leichnetz and Astruc 1975) and direct access of PFC fibers to the MVT was shown in the squirrel monkey (Leichnetz and Astruc 1976). Nauta was aware of the importance of the connections to what he called the “limbic midbrain” and described a reciprocal connection via an uninterrupted continuum of subcortical grey matter. His forebrain connection went from the basal telencephalon via the hypothalamus as far as the paramedian mesencephalon extending into the isthmus rhombencephali. The mesencephalic part comprises the VTA, the dorsal raphe nucleus, the ventral half of tegmental grey matter, the median raphe nucleus and the dorsal and ventral nuclei of Gudden (Nauta 1958). It is interesting to note that the projection pathways of most of these structures is identical with the mfb (Olszewski and Baxter 1982; Allen and Hopkins 1989; Morgane et al. 2005) or part of the greater MFB system (imMFB) (Coenen et al. 2011, 2012, 2018b).

**Fig. 14:**
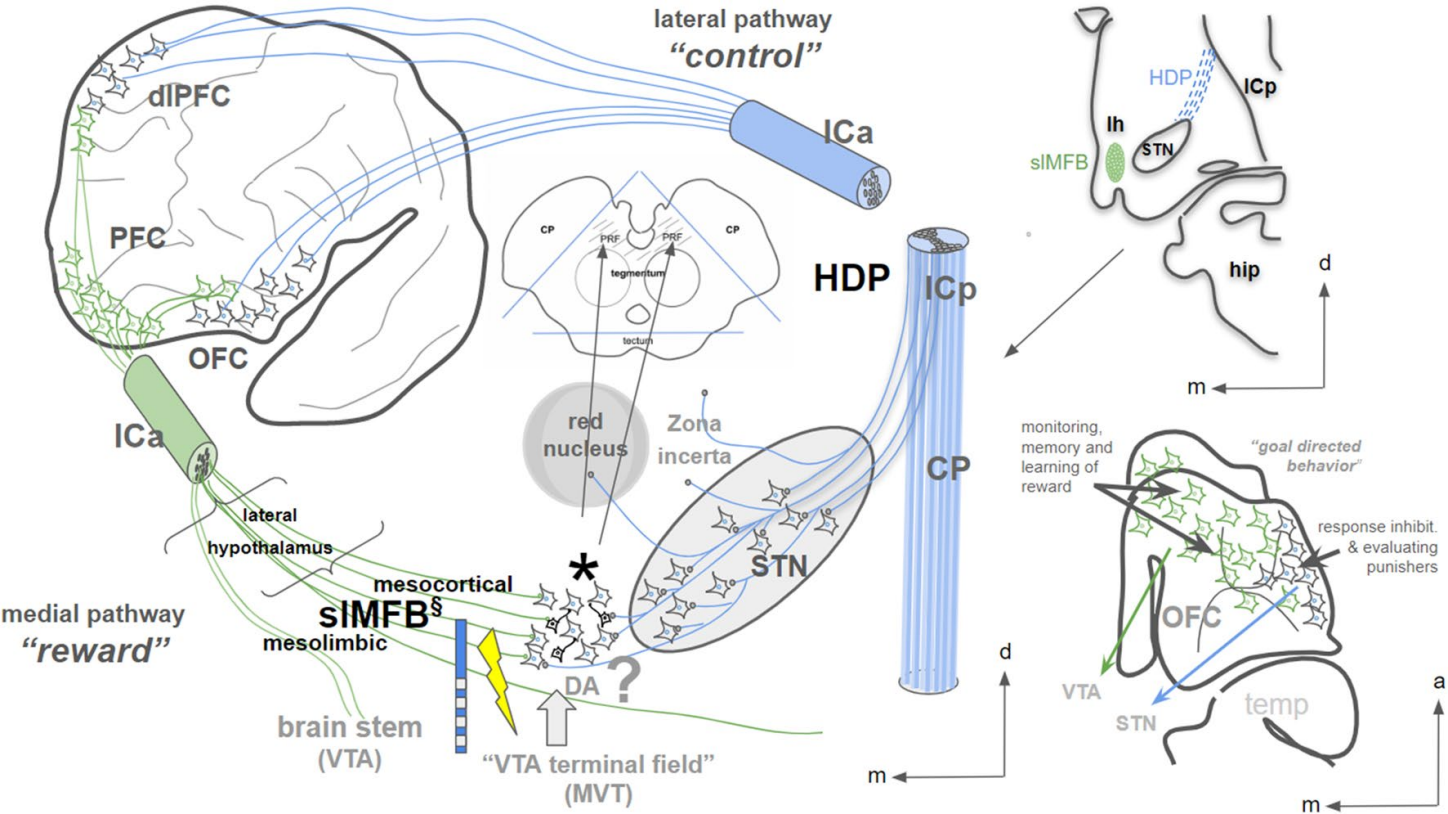
Conceptual cartoon showing the medial pathway (slMFB, green) and the lateral pathway (HDP, blue) in coexistence. Emotional cues, memory of emotions and learning are represented in the medial OFC and the ventromedial PFC (not shown). Response inhibition to emotional cues is realized via the lateral OFC and its hyperdirect projections to the STN (“limbic hyperdirect pathway”). The functional role of the orbitofrontal cortex (OFC) is essentially similar to the description (Kringelbach and Rolls 2004; Kringelbach 2005). §, the slMFB is potentially a bi-directional pathway and is only shown as a descending pathway here. It has direct access to the mesencephalic ventral tegmentum; the wiring pattern and the projections of GABA-ergic interneurons in the region are not fully clear, but an axonal termination field (*) in the lateral VTA just medial to the STN has been described. ?, the role of Dopamine (DA) in the region is not fully clear. *slMFB* superolateral branch of the medial forebrain bundle; *VTA* ventral tegmental area; *PRF* prerubral field (field H of Forel), hatched region in midbrain inset represents mesencephalic ventral tegmentum; *ICa* internal capsule anterior limb; *ICp* posterior limb; *lh* lateral hypothalamus; *CP* cerebral peduncle; *OFC* orbitofrontal cortex; *STN* subthalamic nucleus; *HDP* hyperdirect pathways; *PFC* prefrontal cortex; *dlPFC* dorsolateral; *m* medial; *a* anterior; *d* dosal; *hip* hippocampus

**Fig. 15:**
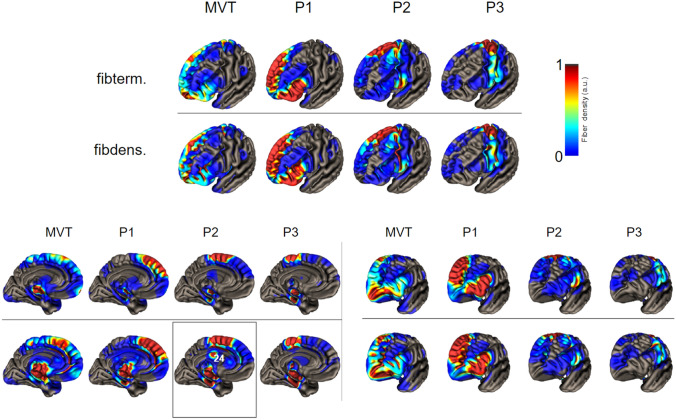
Frontal cortical origins of projections to the midbrain derived from HCP template (*n* = 100). Comparison of fiber terminals and fiber densities. Results are similar, BA 24 is pronounced in fiber densities for P2

Most of the DBS work, related to psychiatric diseases (especially OCD) nowadays focuses on the subthalamic nucleus. However, the greater ventral tegmental midbrain and the adjacent hypothalamus are by no means uncharted (Elias et al. 2020). The posteromedial hypothalamus is adjacent anteriorly and is a DBS target for cluster headache (Fontaine et al. 2010; Chabardès et al. 2016) and aggressiveness (Franzini et al. 2012; Torres et al. 2020). The region itself has been scrutinized with lesioning and stimulation surgery already in the late 1960s and is still referred to as an ergotropic region or the triangle of Sano (Sano 1962; Sano et al. 1966, 1970). For our discussion, the region of interest in the ventral mesencephalic tegmentum is situated in front of the red nucleus and medial to the STN, namely the *pre-rubral field* (PRF) (Fig. 12), which is part of Forel’s field H (Forel 1877; Spiegel et al. 1964). Self-stimulation in the ventral mesencephalic tegmentum is rewarding and activating (Wise and Bozarth 1984) indicating a clear relationship to the reward system. In humans, the region has been scrutinized for its side effect spectrum in stimulation experiments (Sano 1962; Spiegel et al. 1964) and oculomotor and autonomous effects have been described. Similar effects have been seen during slMFB DBS (Coenen et al. 2018a). In non-human primates, a *VTA terminal field* of OFC fibers has been described (An et al. 1998; Öngür et al. 1998; Price 2006). In this context, we would like to point out once more that this region just medial and outside the STN (Fig. 14) produces strong anti-depressant and anti-OCD effects upon stimulation with the DBS technology (Coenen et al. 2011, 2016, 2018a; Tyagi et al. 2019) and has lead to hypomania upon co-stimulation during STN DBS (Coenen et al. 2009). One mode of action of the DBS technology is the antidromic activation of the axonal terminals which show a much lower activation threshold than fibers of passage (Gunalan et al. 2018; Bower and McIntyre 2020). It is therefore a plausible analogy that DBS of the slMFB in part is effective because of the modulation of terminating axons in the lateral VTA termination field with antidromic activation of these OFC descending fibers with an activity change in the OFC as a result. Such antidromic effects of stimulation in the lateral VTA have already been found in animal experiments (Settell et al. 2017).

### Small fiber tracts

The target region for slMFB DBS is situated in a narrow region with a plethora of smaller fiber tracts in its vicinity. It has been debated (Haber et al. 2020) that the antidepressant (and anti-OCD) efficacy originates from the co-stimulation of such microstructures. The here used enhanced resolution in the ex-vivo specimen and the transfer into the MNI environment helps to identify many of these fiber tracts (Fig. 11). A simulation of the volumes of activated tissues (VAT) of previous responders and non-responders in two trials of slMFB DBS for in major depression (Schlaepfer et al. 2013; Coenen et al. 2019a,b) does not underpin such a mechanism. There might be an occasional co-stimulation of the red nucleus or the STN in both responders and non-responders in our analysis here, but these do not appear to be consistent findings. It has to be stated—though—that we have here used only a single anatomical high resolution data set (albeit in MNI space) to analyse this problem, exemplarily. Therefore, these results must be interpreted with caution.

### Pertinent image aggregation studies on DBS in OCD

Simulation studies on OCD have reported the aggregation of image data (dMRI fiber tractography and electrode positions) from DBS patient cohorts which were implanted in distinct target regions (ALIC; VC/VS; amSTN) at different institutions. dMRI information was augmented from the HCP repository. The goal of these studies is the identification of a “common tract” which upon stimulation explains anti-OCD efficacy (Smith et al. 2020; Vlis et al. 2020; Li et al. 2020). The identified pathway appears to be very robust across these studies. It represents a connection between the most ventral part of the ICa and—allegedly—the STN. The pathway structure is typically identified as (limbic) HDP (Vlis et al. 2020; Li et al. 2020). According to the observations of this contribution, it is striking that the tractographically described fibers in all these studies pass *medial to the STN* and on their way down to the brain stem traverse the lateral hypothalamus. However, HDP fibers should be located and enter the STN laterally, subserving the structure out of CP. Figure 8 shows that a medial preference of fiber tracts cannot be explained as an effect of the tractography (“false positives''), since in principle, seeding in the most ventral part of ICa leads to a fiber depiction straddling the nucleus both medial and lateral. The result of a medial to the STN located “common tract” is therefore more likely driven by the actual position of stimulated contacts and adjacent simulated volumes of tissue activation in the amSTN. Tyagi et al. have published an important contribution with *n* = 6 patients suffering from OCD who were implanted with two sets of DBS electrodes each in VC/VS and amSTN. In their thorough tractographic workup of patient individual dMRI data they found that the hotspot of stimulation (for amSTN) across their patients was located in the white matter medial to the amSTN (Fig. 2 in their work, “The average amSTN VTA was centered on the anterior-inferior medial border of the STN *spreading into the ventral tegmental area*.”) (Tyagi et al. 2019). The described amSTN hotspot was already found to be congruent with the slMFB target region (Coenen et al. 2020). It is in this respect possible that a DBS electrode in the amSTN might have its most effective contact outside and medial to the amSTN, a region which has been named the medial STN region (MSR) or “limbic cone of the STN” in the macaque (Haynes and Haber 2013) (see discussion above) but—in our eyes—represents the VTA termination zone of descending OFC fibers (see discussion above). According to the above reasoning, we conclude that the reported “tract target” or “common tract” does not represent the limbic hyperdirect pathway (as part of the control network) as has been stated (Vlis et al. 2020; Li et al. 2020). It is indeed representing the co-existing slMFB and therefore additionally belongs to a related but distinct functional system, the reward network (Coenen et al. 2020).

### Limitations

There are several limitations of this study. The long-range (HCP) tractography was performed in group space. Averaging effects of the dMRI data might lead to false positive as well as false negative findings. However, the tracts we describe here have been already reported on individual level elsewhere (Coenen et al. 2018b; Hosp et al. 2019), thus, we followed here the group approach due to its simplicity and quality of streamline reconstructions. The dichotomy of fiber tracts with strictly lateral trajectories for HDP is derived from the literature and cannot be proven by the dMRI tractographic technology. Therefore, we cannot be sure about connections between the STN and the MVT. The results, however, certainly make sense in comparison with previous studies. Using only a single histological specimen might be regarded as too low. However, the definition of a medial and lateral pathways in the different templates (long range, *n* = 80 HCP; short range *n* = 1 ex-vivo) showed congruent results with the short range information underpinning the results of long range. Especially small fiber tracts like here displayed in the midbrain might show an extensive amount of subject variability (Grisot et al. 2021). As an example the retroflex fascicle might not always be distant to the VAT like shown in the MNI analysis. This aspect is the focus of our future research. Tractography in transition areas between white matter and deep gray matter nuclei (like the STN), which contain high iron deposits, have to be handled with care. The STN shows usually only very subtle diffusion anisotropy, which must not be over interpreted. Diffusion MRI-based tractography cannot make any claims about fiber bundles terminals nor about synaptic patterns.

While registration of the high resolution MRI to MNI space is rather accurate, the registration of histological specimens is rather difficult due to its 2D nature and strong disruptions of the tissue slice. Inaccuracies are in the range of millimeters. However, as individual variations are typical in the same range, and we are trying to make statements on the group level, such imprecision is disturbing but does not question our general conclusions.

## Conclusion

The combination and utilization of long-range and short-range (ex-vivo, high resolution) fiber tractography in the MNI system is feasible. This approach appears to be helpful to investigate regions of interest at higher resolution while not surrendering long-range tractographic capabilities. A larger number of small fiber tracts are located in the proximity to the defined midbrain stimulation region of slMFB DBS for major depression and OCD. Despite this proximity, those structures do not appear to be consistently co-stimulated during slMFB DBS. We have for the first time described parallel pathways to the STN and to the mesencephalic ventral tegmentum (MVT) and separated them with DTI tractographic methods in a long-range/short range combined approach. Based on the new results of this contribution we conclude that fiber pathways to the STN and the MVT coexist and have diverging lateral (posterior capsular/peduncular) and medial (trans-hypothalamic) anatomical routes and physiological functions. These fiber pathways are the hyperdirect pathways and the superolateral medial forebrain bundle. A direct PFC to MVT access as a paralleling structure to cortico-subthalamic projections was predicted already by Nauta based on degeneration and silver impregnation studies. The most ventral structure residing in the anterior limb of the internal capsule (ICa) is the slMFB, connecting the OFC to the prerubral field and here specifically to a lateral VTA termination field and then further to the remaining MVT. The HDPs role in motor-control and regulation of behaviour (control network) as a reaction to external (environmental) and internal (emotional, cognitive, memory) cues is ideally fulfilled with a lateral route and a dominant lateral orbitofrontal origin. The slMFB’s role in the reward network has no control function. It is largely confluent with the reward system and therefore signals pleasant or aversive stimuli while at the same time driving motivational states to obtain pleasant and avoid aversive ones. The results reported here shed new light on the ongoing discussion concerning the role of the slMFB in contrast to the limbic hyperdirect pathway in DBS targeting for depression and OCD. The slMFB was demonstrated clearly as a medial cortico-tegmental projection co-existing with a lateral hyperdirect cortico-subthalamic pathway. This study suggests that the slMFB can be the commonly stimulated structure in effective DBS for OCD and depression.

## Figures and Tables

**Fig. 16:**
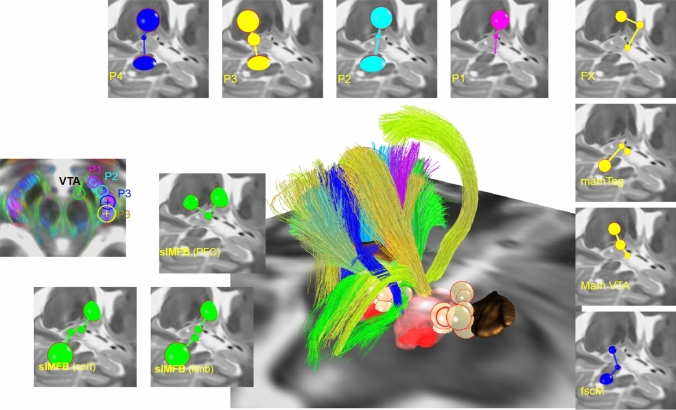
Visualization of the selection templates used for bundle selection. Typically, a template consists of one defining ‘bottleneck’ sphere, which is rather tight, and two rather large spherical volumes of interest defining the rough direction of the bundle of interest. Pink spheres in the central image represent simulated activation volumes from responders in FORESEE and FORESEE II trials for treatment-resistant major depression

**Fig. 17:**
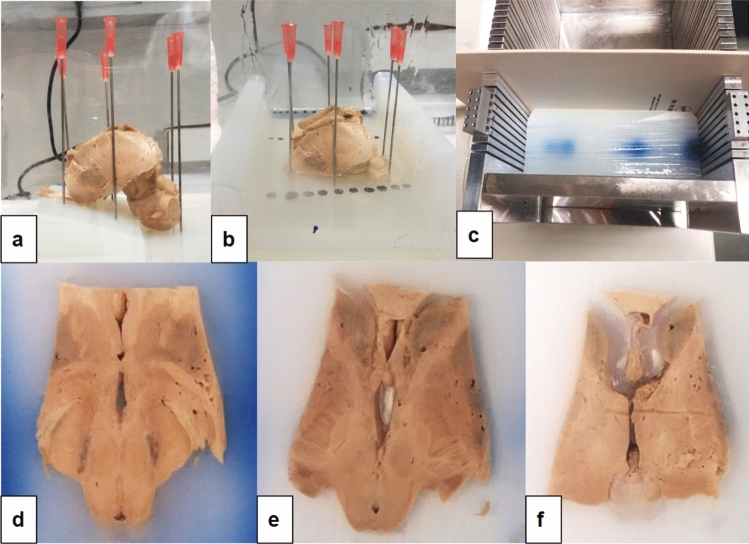
**a**, **b**, Pictures taken during the preparation procedure for cutting the midbrain specimen. The specimen is placed in a Perspex box (20 × 20 × 20 cm), with a titanium base and four mountable/ removable walls. **b** Approximately 2 L of a 5% agar solution was poured into the box, and when it started to set, the brain was placed on the agar base and the anterior commissure—posterior commissure was horizontally aligned; **c** the hardened agar block is placed in a box with rails of 5 mm spacing. **d**–**f** Examples of ACPC-parallel cuts of 5 mm thickness (macro, unstained)

**Table 1:**
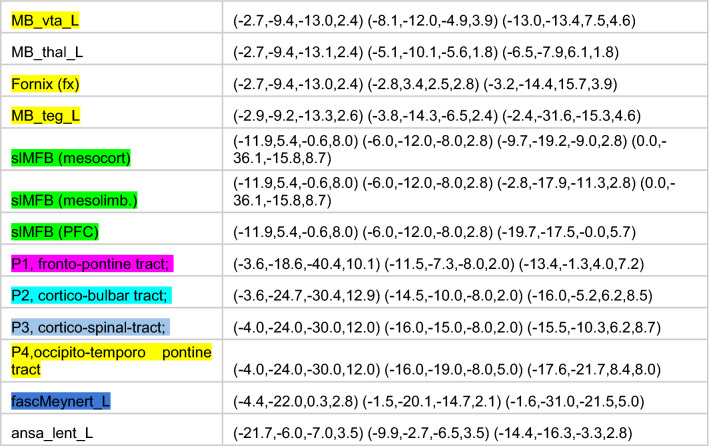
The exact definitions of all selection templates in MNI space. Each template consists of a list of spherical volumes of interest. Each spherical volume is defined by four numbers: (MNI x, MNI y, MNI z, radius)

## Appendix

See Figs. ^15^, 16, and 17.

See Table 1.

## Abbreviations

ALIC: Anterior limb of internal capsule (DBS target)
amSTN: Anteromedial STN
bc: Brainstem/cerebellum bundle of (motorMFB)
CP: Cerebral peduncle
DA: Dopamine
dlPFC: Dorsolateral PFC
dMRI: Diffusion-weighted magnetic resonance imaging
Glu: Glutamate
H: Forel’s field H
HDP: Hyperdirect pathway
ICa: Internal capsule, anterior limb
ICp: Internal capsule, posterior limb
imMFB: Inferomedial branch of the MFB (human primate)
lHDP: Limbic HDP
lh: Lateral hypothalamus
mc: Mesocortical part (of slMFB)
ml: Mesolimbic part (of slMFB)
lOFC: Lateral OFC
mb: Mammillary body bundle (of motorMFB)
mfb: Medial forebrain bundle (rodent)
MFB: Medial forebrain bundle (human primate)
MSR: Medial STN region
MVT: Mesencephalic ventral tegmentum
mOFC: Medial OFC
NA: Noradrenaline
OFC: Orbitofrontal cortex
pfc: Pfc bundle (of motor MFB)
PFC: Prefrontal cortex
PRF: Pre-rubral field
slMFB: Superolateral MFB
SN: Substantia nigra
STN: Subthalamic nucleus
VC/VS: Ventral capsule, ventral striatum (DBS target)
VAT: Volume of activated tissue (simulation)
vmPFC: Ventromedial PFC
VTA: Ventral tegmental area

## Declarations

### Disclaimer

This paper represents a conceptual view which is only in part based on own treated patients but on the combined evaluation of a normative cohort (HCP). The imaging information therefore is not intended for surgical approaches, stimulation planning or steering which—in our view—should always be based on individual imaging.
